# Serine Acetyltransferase from *Pseudomonas aeruginosa*: Distinctive Features, Pleiotropic Roles, and Therapeutic Potential

**DOI:** 10.3390/ijms27115091

**Published:** 2026-06-04

**Authors:** Francesco Guggino, Sarah Hijazi, Rebecca Martedì, Valeria Buoli Comani, Jole Maria Lucia D’Angelo, Omar De Bei, Giannamaria Annunziato, Marco Pieroni, Gabriele Costantino, Stefano Bettati, Marialaura Marchetti, Emanuela Frangipani, Barbara Campanini

**Affiliations:** 1 Interdepartmental Center Siteia, University of Parma, 43124 Parma, Italy; francesco.guggino@studenti.unipr.it (F.G.); stefano.bettati@unipr.it (S.B.); 2 Department of Biomolecular Sciences, University of Urbino Carlo Bo, 61029 Urbino, Italy; 3 Interdepartmental Center Biopharmanet-TEC, University of Parma, 43124 Parma, Italy; valeria.buolicomani@unipr.it (V.B.C.); barbara.campanini@unipr.it (B.C.); 4 Department of Food and Drug, University of Parma, 43124 Parma, Italy; jolemarialucia.dangelo@unipr.it (J.M.L.D.);; 5 Department of Medicine and Surgery, University of Parma, 43125 Parma, Italy; omar.debei@unipr.it (O.D.B.)

**Keywords:** cysteine biosynthesis, serine acetyltransferase, CysE, truncated CysE, antimicrobial resistance, virulence, metabolic inhibitors

## Abstract

Cysteine biosynthesis is increasingly recognized as a critical determinant of bacterial virulence, highlighting this pathway as a promising reservoir of novel antimicrobial targets. In *Pseudomonas aeruginosa*, however, the molecular basis of cysteine production has only recently begun to emerge. Here, we identify PA3816 as the major *P. aeruginosa* serine acetyltransferase (PaCysE), the enzyme responsible for generating the activated serine intermediate that feeds *O*-acetylserine sulfhydrylase-mediated cysteine synthesis. Through a combination of biochemical and genetic approaches, we demonstrate that PaCysE efficiently catalyzes L-serine acetylation *in vitro*, and in turn, deletion mutants exhibit cysteine auxotrophy, underscoring its essential contribution to *O*-acetylserine production. Notably, PaCysE is less sensitive to feedback inhibition by cysteine and does not appear to form the canonical cysteine synthase complex, suggesting a regulatory architecture that diverges from well-characterized orthologs. Loss of PaCysE function has broad physiological consequences, including enhanced biofilm formation, reduced pyocyanin production, and attenuated infectivity in an animal model, linking cysteine biosynthesis directly to pathogen fitness. Finally, we identify a thiazole derivative that inhibits PaCysE activity (IC_50_ ≈ 30 µM) and suppresses bacterial growth in a cysteine-dependent manner, providing a proof-of-concept for therapeutically targeting this pathway.

## 1. Introduction

The cysteine biosynthetic pathway in bacteria represents a central metabolic crossroad that balances the synthesis of cofactors, siderophores, and reducing agents. Due to its role and to its intrinsic toxicity, L-cysteine (L-Cys) plays a fundamental role in bacterial fitness, and its intracellular concentration must be finely controlled. Sulfur assimilation and L-Cys biosynthesis in bacteria have been primarily studied in *Salmonella enterica* serovar Typhimurium, *Haemophilus influenzae*, and *Escherichia coli*, but increasing evidence of their involvement in infection, resistance mechanisms and biofilm formation is driving growing interest in other microorganisms [1,2,3,4,5]. L-Cys synthesis originates from the intersection between two pathways that involve sulfur reduction and the production of an activated form of L-serine (L-Ser). In particular, the last two enzymatic steps are catalyzed by L-Ser *O*-acetyltransferase (SAT, CysE, EC: 2.3.1.30)—which synthesizes *O*-acetyl-L-serine (OAS) from L-Ser and acetylcoenzyme A (AcCoA)—and *O*-acetylserine sulfhydrylase, generally present in at least two isoforms (OASS-A, CysK, and OASS-B, CysM), responsible for L-Cys synthesis from OAS and bisulfide (EC 2.5.1.47) or thiosulfate (EC 2.5.1.144) [6].

CysE is an oligomeric protein belonging to the *O*-acyltransferase family, that presents a triangular cross-section characterized by a left-handed β-helix folding; this region in the monomer drives the assembly of a homotrimer, in which the interfaces between subunits generate the catalytic clefts. The N-terminus consists of an α-helical core with varying length across bacterial species. Typically, this region is shorter in Gram-positive bacteria and longer in Gram-negative species; consequently, these isoforms are commonly referred to as truncated and full-length, respectively. The N-terminal region participates in the association of two trimers into a homohexamer. Accordingly, truncation could in principle affect the assembly underlying distinct regulatory mechanisms, favoring a trimeric state over the hexameric one [7,8].

Many bacterial CysE orthologs have been characterized to date [7,9,10,11,12,13,14,15,16,17,18,19]. Among these, the most comprehensive characterization has been reported for *E. coli* CysE (EcCysE). Its catalytic mechanism is generally described as a random-ordered reaction, in which either AcCoA or L-Ser may bind first, leading to the formation of a ternary complex [11]. The main regulatory mechanism that controls CysE activity is feedback inhibition by L-Cys. Notably, this mechanism heavily limits the amount of L-Cys that can be obtained by bacterial fermentation [20]. The mechanism that controls inhibition by L-Cys is interesting: L-Cys competes with L-Ser for active site binding, but its acetylation is prevented by the intrasteric inhibition exerted at the AcCoA binding site by the flexible C-terminal sequence of the enzyme [10]. Indeed, mutations of the C-terminal sequence relieve L-Cys inhibition to different extents [20]. In several species, CysE C-terminus further tunes L-Cys biosynthesis by the formation of the Cysteine Synthase Complex (CSC) engaging the CysK active site. This interaction has been proven to elicit significant conformational dynamics in EcCysE structure, with long-distance allosteric changes [21]. CysE catalytic efficiency is thus increased when the enzyme is recruited in the complex and CysK is partially inhibited; at the same time, CSC is stabilized by bisulfide, while OAS promotes its dissociation [22,23]. It has been demonstrated that the metabolic node managed by CysE has a pervasive importance in bacteria, with an extended impact on several metabolic pathways other than L-Cys biosynthesis. Indeed, the reaction catalyzed by CysE lies at the crossroad between one-carbon and sulfur metabolism, with L-Ser derived from glycolysis participating in many vital processes like phospholipids and nucleotide biosynthesis. Furthermore, the product OAS spontaneously isomerizes to *N*-acetylserine (NAS), which is the inducer that controls the expression of the cysteine regulon in *E. coli* and *S.* Typhimurium [6].

Experimental studies on *cysE*-deleted strains of *Providencia stuartii*, *E. coli* and *S.* Typhimurium revealed that the lack of enzymatic activity is linked to altered cellular phenotypes, such as enhanced biofilm development and altered redox state, which are relieved by exogenous supplementation of OAS or cysteine [24,25,26]. Accordingly, *Mycobacterium tuberculosis* Δ*cysE* showed an impaired response to oxidative stressors; in addition, the deletion was linked to an altered transcriptional profile regarding genes involved either in the energetic metabolism or iron acquisition [27]. In *Serratia marcescens*, *cysE* contributes to additional physiological functions, as its deletion leads to reduced phospholipase activity and complete loss of swarming motility, phenotypes that are restored by exogenous supplementation with OAS or L-Cys [28]. Involvement of CysE in swarming motility has also been described in *S.* Typhimurium, a bacterial species where CysE has been shown to play a prominent role in a murine model of infection [26,29].

In line with the increasing interest elicited by L-Cys biosynthetic pathway in the search of novel targets to develop antimicrobial compounds, several studies reported medicinal chemistry efforts towards CysE isoforms [1,7,30,31,32,33,34].

Nevertheless, despite the worldwide diffused infection burden and the struggling need to develop new antimicrobials, cysteine metabolism—and in particular CysE functions—has never been studied in detail in *Pseudomonas aeruginosa*. Starting from genome analyses, in this work we validated the assignment of the gene PA3816 from *P. aeruginosa* PAO1 to a SAT/CysE by phenotypic and biochemical analyses. The PA3816-deleted strain is auxotrophic for cysteine and shows increased susceptibility to oxidative stress, altered virulence factors production, enhanced biofilm formation, and reduced virulence *in vivo.* Structural analyses revealed that *P. aeruginosa* CysE (PaCysE) belongs to the N-terminal truncated clade, and shows an extended C-terminus similar to that predicted for *M. tuberculosis* isoform [35]. The results of the enzyme characterization *in vitro* suggest that PaCysE activity is not regulated by CysK, differently than what is observed in other bacterial species [13]. Finally, we screened a small library of compounds previously proposed for *S.* Typhimurium CysE inhibition [32,36], showing promising results for the development of targeting molecules.

## 2. Results and Discussion

### 2.1. Gene Identification and Phenotypic Characterization of the Deletion Mutant

#### 2.1.1. Sequence Identification and Analysis

No enzyme responsible for producing OAS in *P. aeruginosa* has been characterized so far. To identify the corresponding coding gene, we interrogated the BioCyc database (https://biocyc.org/), choosing *P. aeruginosa* PAO1 as the reference genome database and searching for SAT activity. The database returned two results corresponding to the query (Gene-Ontology-Terms Class GO:0009001), specifically annotated as *O*-acetylserine synthase (*cysE*, PA3816) and acetyltransferase (PA2105). We then performed a homology search on Pseudomonas DB (https://www.pseudomonas.com (accessed on 20 March 2026)) using the BLASTP search against *P. aeruginosa* PAO1 strain and as a query the UniProt sequence P29847, corresponding to *S.* Typhimurium CysE (StCysE). By setting 1 × 10^−8^ as the expected value cutoff—which is an estimate of the likelihood of a specific alignment—the two hits returned matched the previous results, with P29847 percentage identities equal to 44% for PA3816 (4.5 × 10^−44^) and 34.8% for PA2105 (6.9 × 10^−23^), respectively. PA3816 gene sequence is located on the negative strand and co-occurs with a putative RNA methyltransferase (PA3817), whereas the downstream genes constitute the *isc* operon, responsible for the synthesis, regulation and assembly of iron-sulfur clusters. PA2105 encodes a probable acetyltransferase and interestingly co-occurs with a probable cysteine synthase (PA2104). In other bacterial genomes, *cysE* usually does not take part in the *cysK* or *cysM* operons; however, several bacterial and plant species possess more than one SAT isoform working under different regulations, therefore not excluding a possible redundancy of this activity also in *P. aeruginosa* [7,14,37,38].

**Figure 1:**
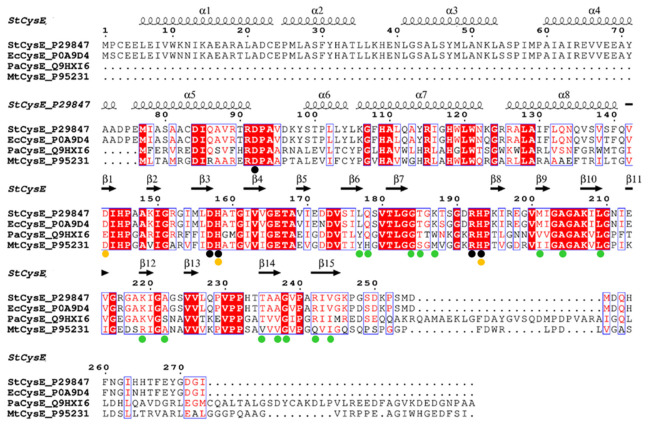
**Multiple alignment of CysE sequences from *S.* Typhimurium (St), *E. coli* (Ec), *P. aeruginosa* (Pa) and *M. tuberculosis* (Mt).** Codes correspond to Uniprot IDs. Green circles indicate the residues involved in AcCoA binding, whereas black ones correspond to residues involved in L-Ser binding; orange circles indicate the residues involved in the catalysis. The secondary structure of *S.* Typhimurium CysE reported on top of the alignment was retrieved from PDB ID 8i09 [33]. The alignment was generated by ClustalX 1.81 [39] and visualized by ESPRIPT3 [40].

Based on the reported pieces of evidence, we primarily focused our attention on PA3816. To further assess the correspondence of PA3816 with a CysE-encoding gene, we aligned its translated sequence to those of known bacterial SATs and looked for conserved binding and catalytic residues. The alignment of the amino acid sequence with the orthologs from *S.* Typhimurium, *E. coli*, and *M. tuberculosis* revealed the overall conservation of the residues involved in L-Ser binding (Figure 1, [10,33]). In particular, kinetic and structural studies on the *E. coli* isoform supported a specific role in the catalysis for His193, Asp143, and His158, the latter playing the role of general base to deprotonate the hydroxyl group of the substrate L-Ser [12]. The two histidine residues are conserved, whereas the aspartate is substituted by a glutamate, conserving the physico-chemical properties of the residue at that position. On the contrary, residues involved in AcCoA binding are only partially conserved. Given the overall sequence conservation, particularly regarding the catalytic residues, we hereafter refer to PA3816 as PaCysE, although still awaiting further physiological and biochemical characterizations (*vide infra*).

To have a first structural glimpse, we used ESPRIPT3 (https://espript.ibcp.fr/ESPript/ESPript/ (accessed on 20 April 2026)) to visualize on the alignment the corresponding secondary structure elements of *S.* Typhimurium. This comparison makes it possible to appreciate that PaCysE presents a truncated N-terminus, lacking four α-helices, and an elongated C-terminal region, similarly to the *M. tuberculosis* enzyme (Figure 1). Curiously, PaCysE represents—with few other cases—quite an exception among γ-Proteobacteria, which mostly possess a full-length enzyme [7].

#### 2.1.2. Investigation of the Physiological Role of CysE in *P. aeruginosa*

**Figure 2:**
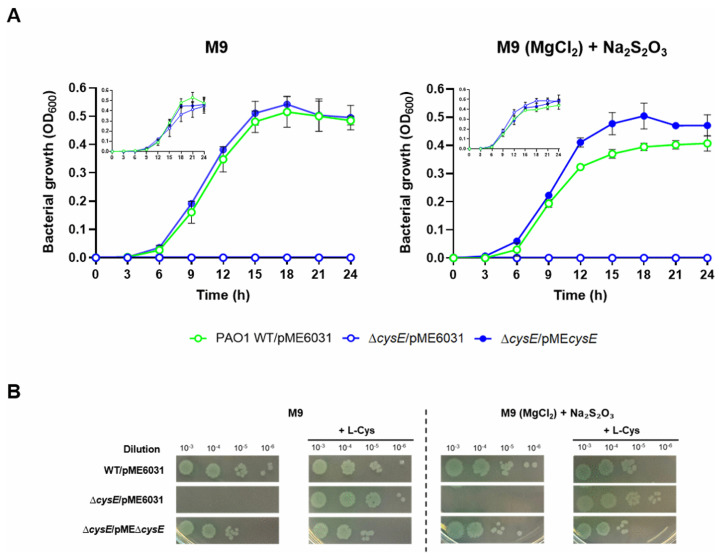
**Phenotypic characterization of *P. aeruginosa* Δ*cysE*.** (**A**) *P. aeruginosa* PAO1 (green) and its isogenic Δ*cysE* mutant (blue) were cultured in liquid M9 or M9 containing Na_2_S_2_O_3_ (0.5 mM) as the alternative S-source. Growth in media supplemented with L-Cys (40 μg/mL) is shown in insets, using the same axes scale reported in the main figures. Circles indicate the presence of the empty plasmid pME6031 while filled circles indicate the presence of pME*cysE* in the Δ*cysE* mutant. Growth was measured by turbidimetry (OD_600_) over time. Each value is the average of three biological replicates each performed in duplicate ± standard deviation. (**B**) Colony growth of *P. aeruginosa* PAO1 (WT) and its isogenic Δ*cysE* mutant carrying the empty plasmid pME6031 or pME*cysE*, as indicated. Strains were grown on solid M9 or M9 (MgCl_2_) + Na_2_S_2_O_3_ supplemented or not with L-Cys. Stationary-phase cultures were normalized to OD_600_ = 1, and 5 μL of the indicated dilutions were spotted onto the plates, which were then incubated for 24 h at 37 °C. The images are representative of three independent experiments yielding similar results.

To investigate the physiological role of CysE, an in-frame deletion mutant was constructed in *P. aeruginosa* PAO1, generating the Δ*cysE* strain. The growth of this mutant was evaluated in the chemically defined M9 medium containing sulfate or thiosulfate as the sole sulfur source. In the case of thiosulfate, MgSO_4_ was substituted with an equimolar concentration of MgCl_2_. While *P. aeruginosa* WT was able to grow in both media, *P. aeruginosa* Δ*cysE* exhibited an auxotrophic phenotype, unable to sustain growth under the tested conditions, with OD_600_ values remaining at baseline across all examined time points (Figure 2A). To determine whether the growth defect of *P. aeruginosa* Δ*cysE* was due to the lack of cysteine, Δ*cysE* cultures were supplemented with L-Cys at 40 µg/mL [41]. Under this condition, the mutant exhibited a growth profile comparable with that of the WT (Figure 2A, insets). As a further validation, the *P. aeruginosa* Δ*cysE* mutant was genetically complemented by providing *cysE in trans* on the pME*cysE* plasmid. The wild-type phenotype was restored in *P. aeruginosa* Δ*cysE*/pME*cysE*, whereas the presence of the empty plasmid pME6031 did not alter the growth behavior of all tested strains (Figure 2A). Overall, these results demonstrated that CysE is essential for both sulfate- and thiosulfate-dependent L-Cys biosynthesis in *P. aeruginosa*, consistent with its supposed role in catalyzing the formation of OAS, the first committed step in cysteine biosynthesis.

In parallel, we also assessed the colony growth of the WT and Δ*cysE*, on the above-mentioned media solidified with 1.5% agar (Figure 2B). Consistent with the observations in liquid broth, Δ*cysE* displayed L-Cys auxotrophy, showing no detectable growth on solid media (either with sulfate or thiosulfate) up to 24 h post-inoculation. In contrast, supplementation of the media with L-Cys or genetic complementation restored the growth of the Δ*cysE* mutant (Figure 2B). The marked auxotrophy observed in the *P. aeruginosa* Δ*cysE* mutant is in line with similar phenotypes reported in both Gram-positive and Gram-negative bacteria, including *S.* Typhimurium, *S. marcescens*, *E. coli*, *Staphylococcus xylosus*, and *Corynebacterium glutamicum* [28,42,43,44,45] in which *cysE* mutants are unable to grow in minimal media unless L-Cys is supplied.

#### 2.1.3. The Lack of CysE Leads to Increased ROS Production

**Figure 3:**
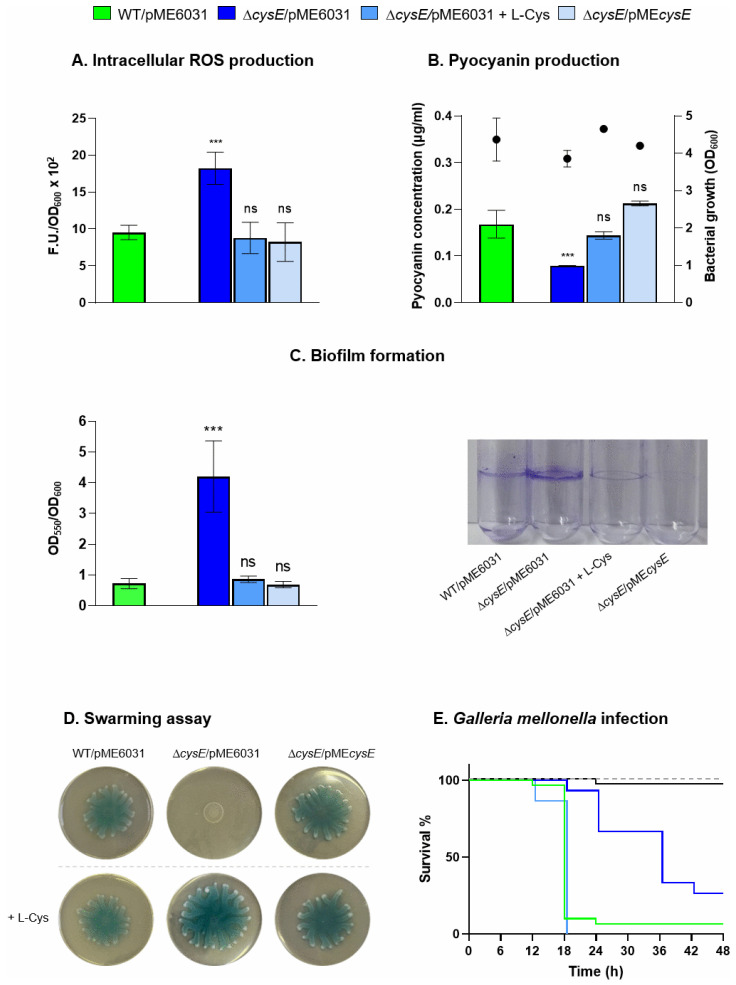
**Role of *cysE* in *P. aeruginosa* virulence.** (**A**) Intracellular ROS levels (expressed as Fluorescence Units, F.U.) were determined using the fluorescent dye 2′,7′-dichlorodihydrofluorescein diacetate (H_2_DCFDA) in the indicated strains grown to the mid-exponential phase (OD_600_ ≈ 0.6) in LB supplemented or not with 40 µg/mL L-Cys at 37 °C, under vigorous shaking. (**B**) Pyocyanin production by the indicated strains grown in LB with or without L-Cys was quantified by measuring the absorbance of the acidified supernatant at 520 nm. (**C**) Biofilm formation by the indicated strains was assessed after 24 h of incubation at 37 °C in 96-well plates (left) or glass tubes (right) using a crystal violet assay. Images are representative of three independent experiments with similar results. Values in (**A**–**C**) represent the means +/− SD of three independent assays. Statistical analysis was performed using one-way ANOVA with Tukey’s multiple comparisons test. Differences between the WT and Δ*cysE* (either complemented or not with pME*cysE* or grown with L-Cys supplementation) were considered statistically significant if *p* < 0.001 (***), or not significant (ns) if *p* ≥ 0.05. (**D**) Swarming motility was evaluated by spotting 10 µL of stationary-phase bacterial cultures onto solid Nutrient Broth medium containing 0.6% agar, followed by incubation at 37 °C for 18 h. Images are representative of three independent experiments with similar results. (**E**) Kaplan–Meier survival curves of *Galleria mellonella* larvae infected with 10 µL of a 10^3^ CFU/mL suspension of the indicated *P. aeruginosa* strains or injected with saline (negative control) and incubated at 37 °C for 48 h. Each experiment included 30 larvae per group. Statistical analysis was performed using the log-rank (Mantel-Cox) test. Differences between the WT and Δ*cysE*, as well as between the Δ*cysE/*pME*cysE* and Δ*cysE* infected larvae, were considered statistically significant (*p* value < 0.05).

Reactive oxygen species (ROS) generated during aerobic metabolism can damage macromolecules belonging to essential cellular components, including DNA, proteins, and lipids. To survive, bacteria must detoxify ROS through antioxidant enzymes and redox buffers, with glutathione (GSH)—a cysteine-derived antioxidant—serving as the predominant redox buffer [2]. Accordingly, we sought to determine how disruption of L-Cys biosynthesis—here achieved through the *cysE* deletion—could affect intracellular ROS levels in *P. aeruginosa*. To this aim, bacteria harvested from the mid-log growth phase were analyzed for ROS production using the 2′,7′-dichlorofluorescein diacetate (DCFH-DA) fluorescent probe. As shown in Figure 3A, Δ*cysE* exhibited approximately a twofold increase in ROS production compared with the WT, indicating an elevated oxidative stress level. Supplementation of the medium with L-Cys or genetic complementation of the Δ*cysE* mutant restored tolerance to oxidative stress (Figure 3A). These results are consistent with previous observations in *M. tuberculosis* and *Brucella ovis*, where disruption of *cysE* increased sensitivity to oxidative stress, highlighting the importance of CysE in maintaining redox homeostasis [3,27].

#### 2.1.4. Pyocyanin Production Is Reduced When CysE Is Missing

Disruption in the redox status has been linked to the expression of virulence factors, including pyocyanin, a pro-oxidant, blue-green phenazine pigment associated with infection in *P. aeruginosa* [46,47,48]. This prompted us to investigate pyocyanin production in the Δ*cysE* mutant. To this end, bacteria were cultured in Luria-Bertani (LB) with vigorous shaking, and pyocyanin was extracted using chloroform followed by re-extraction into 0.2 N HCl. The amount of pyocyanin produced by Δ*cysE* (0.08 µg/mL) was significantly lower than that produced by the WT (0.17 µg/mL), while supplementation with L-Cys or genetic complementation restored pyocyanin production, with no significant difference observed between the WT and the complemented Δ*cysE* strain (Figure 3B), suggesting a role of CysE in its production. The reduced intracellular L-Cys levels in the Δ*cysE* mutant may indeed lower GSH availability, which might expose bacterial cells to pyocyanin-mediated self-toxicity [48]. In this context, it is plausible that compensatory mechanisms are activated to mitigate oxidative damage, including downregulation of pyocyanin production itself. Although pyocyanin is known to be tightly regulated by a complex network involving quorum sensing systems (Las/Rhl/PQS), redox sensing regulators such as (SoxR), and metabolic/iron-dependent signals [49,50,51], which collectively tune phenazine biosynthesis in response to cell density and intracellular redox status, other mechanisms might also be involved. Notably, the cysteine-rich metallothionein PmtA has recently been identified as a positive regulator of pyocyanin production in *P. aeruginosa* [52]. It is tempting to speculate that when CysE is missing, L-Cys depletion might in turn reduce PmtA availability, causing a downregulation of pyocyanin production.

#### 2.1.5. The Lack of CysE Leads to Increased Biofilm Formation

Since the redox status has been shown to influence bacterial lifestyle transitions from planktonic to biofilm [53], we assessed whether cysteine deficiency could alter biofilm formation in *P. aeruginosa*. Strains were grown in 96-well plates for 24 h in M63 medium, a standard medium for biofilm assays in *P. aeruginosa*, after which biofilm formation was quantified using the crystal violet assay [54]. While the WT exhibited robust planktonic growth, the Δ*cysE* mutant showed impaired planktonic growth but preferential surface attachment to the walls of the microtiter plate (Figure 3C). Consistent with this observation, a more pronounced biofilm ring was visible for the Δ*cysE* mutant also in glass tubes (Figure 3C). This effect was reversed upon supplementing the medium with L-Cys or genetic complementation of Δ*cysE* with a wild-type copy of *cysE* gene (Figure 3C). Interestingly, a similar behavior has previously been documented in an *E. coli cysE* mutant [24]. The mechanism linking CysE and biofilm formation has not been further elucidated; however, the observed shift toward a sessile lifestyle under cysteine limitation might be associated with increased intracellular levels of cyclic-di-GMP, a second messenger that promotes biofilm formation and represses motility in *P. aeruginosa* [55,56]. Interestingly, disruption of L-Cys biosynthesis has recently been linked to the accumulation of cyclic-di-GMP, hence to an enhanced sessile lifestyle in other bacterial species (i.e., *Azospirillum brasilense* [57]), further supporting this hypothesis.

#### 2.1.6. CysE Contributes to *P. aeruginosa* Swarming Motility

As motility is an important virulence factor for *P. aeruginosa* [58], we investigated how disruption of *de novo* L-Cys biosynthesis would affect its flagellum-mediated motility. For that, *P. aeruginosa* strains were cultured on NB (Nutrient Broth) medium containing 0.6% agar to facilitate surface motility. Results demonstrated that the WT swarmed readily over the 18 h incubation period, forming a thick film of growth over the surface of the agar, whereas the Δ*cysE* mutant failed to migrate beyond the point of inoculation (Figure 3D). As expected, addition of exogenous L-Cys or genetic complementation of the mutant with *cysE* restored the wild-type motility phenotype (Figure 3D). Of note, the reduced swarming motility observed with Δ*cysE* was not due to impaired growth resulting from cysteine auxotrophy, as the mutant Δ*cysE* grew in NB-agar medium (see Figure S1). The observed motility defect may instead be attributable to the reduced functional flagella, which may depend on L-Cys biosynthesis, as previously reported in *S. marcescens*, where mutation of *cysE* results in decreased transcription of flagellar genes, thus impairing swarming motility [28]. Additionally, L-Cys depletion in the Δ*cysE* mutant might reduce the intracellular level of GSH, which has been shown to impair flagellar motility in *P. aeruginosa* [46].

#### 2.1.7. The Lack of CysE Attenuates the Virulence of *P. aeruginosa* in *Galleria mellonella*

The virulence of the Δ*cysE* mutant was finally evaluated *in vivo* using the *G. mellonella* infection model. Notably, the Δ*cysE* strain exhibited a markedly attenuated virulence phenotype, causing approximately 30% larval mortality up to 24 h post-infection, compared to about 90% for the WT (Figure 3E). However, larval mortality in the Δ*cysE* group increased progressively over time but did not reach the levels observed for the WT. Genetic complementation of the Δ*cysE* mutant restored the wild-type phenotype as early as 18 h post-injection, suggesting a role of CysE during infection.

Collectively, these findings indicate that CysE likely plays pleiotropic activities that go beyond cysteine biosynthesis, contributing to the maintenance of redox homeostasis, the regulation of virulence-associated phenotypes, and *in vivo* pathogenicity of *P. aeruginosa*.

### 2.2. Structural and Functional Analysis

#### 2.2.1. Recombinant Protein Expression and Biophysical Characterization

**Figure 4:**
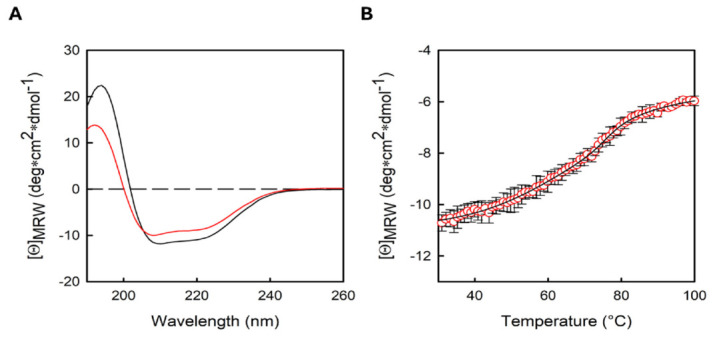
**Structure and stability of PaCysE.** (**A**) Circular dichroism spectra before (black) and after (red) thermal denaturation reported in panel (**B**). (**B**) Dependence of the mean residue ellipticity on the temperature in the 30–100 °C range. The black line through the red data points is the fitting to Equation (3) with a melting temperature of 74 ± 2 °C.

PaCysE was expressed recombinantly in *E. coli* as a fusion protein with a N-terminal Strep-tag in low yields (1 mg/L). After CysE overexpression, the OD_600_ of the induced *E. coli* culture was reproducibly similar to that measured before induction. After cell lysis, more than half of the expressed protein was in the insoluble fraction. Together, these observations likely point to a toxic effect in *E. coli* or to an intrinsic poor solubility of the protein (Figure S2). The final preparation has a homogeneity of more than 95%. The circular dichroism (CD) spectrum in the far-UV of PaCysE is typical for a mixed alpha/beta structure, which is confirmed by the results of the spectra deconvolution (Figure 4A, Table S1). These results are also in very good agreement with the secondary structure content obtained by the analysis of the AlphaFold 3 predicted structure performed by the KCD web server [59] (*vide infra*, Table S1).

The T_m_ calculated on the thermal denaturation curve is 74 ± 2 °C (Figure 4B), a value significantly higher than the mean T_m_ value for a mesophilic protein, which is about 62 °C [60]. This points to a very good thermodynamic stability for this protein that is not fully consistent with the reported marginal stability of *E. coli* ortholog [61]. Furthermore, the spectrum of the solution after completion of the thermal scan still shows spectral features indicative of residual secondary structure (Figure 4A, Table S1).

Most bacterial CysEs characterized to date assemble into hexamers (dimer of trimers); however, the reduced number of α-helices at the N-terminal region of PaCysE, that constitutes the dimerization region of CysE homotrimers, is supposed to correlate with an oligomeric equilibrium favoring the trimeric assembly. To date, no crystallographic structure is available for CysE truncated isoforms [7].

**Figure 5:**
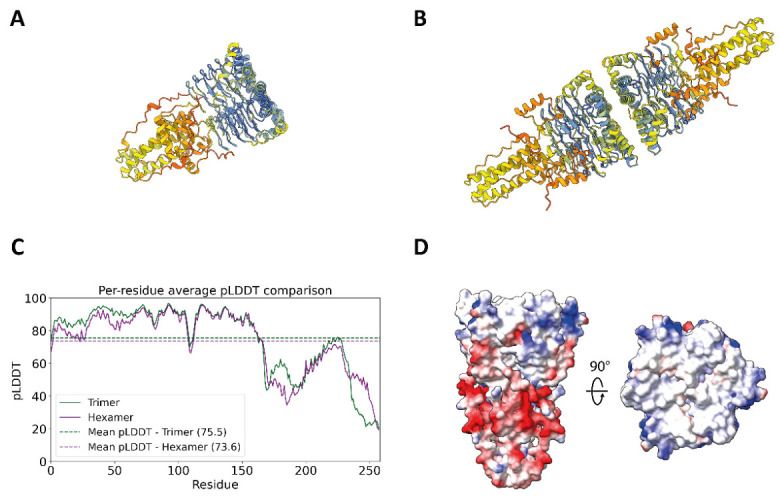
**Structural prediction and per-residue confidence of CysE oligomers.** (**A**) Trimeric model of CysE predicted by AlphaFold3, colored according to the pLDDT score: blue (pLDDT > 90), light blue (90 > pLDDT > 70), yellow (70 > pLDDT > 50), and orange (pLDDT < 50). (**B**) Hexameric model predicted by AlphaFold3, colored using the same pLDDT scale. (**C**) Comparison of the average per-residue pLDDT profiles for the trimeric (green) and hexameric (purple) models. For each oligomer, per-residue pLDDT values were averaged across all constituent chains (three for the trimer and six for the hexamer). Dashed horizontal lines indicate the overall mean pLDDT for each oligomer. (**D**) Electrostatic surface representation of the PaCysE trimer shown in side and top views. Surface potentials are colored according to charge (blue, positive; red, negative; white, neutral).

The low yields of protein expression and purification hampered the determination of the three-dimensional structure of PaCysE by crystallography; we thus used AlphaFold3 [62] to model two oligomeric assemblies, a trimer and a hexamer (Figure 5A,B), and assessed prediction reliability using the predicted Local Distance Difference Test (pLDDT), a per-residue confidence score ranging from 0 to 100, where higher values indicate greater confidence in the predicted atomic positions (Figure 5C). To quantitatively compare the models, we extracted the average per-residue pLDDT of the monomers and plotted the profiles for both assemblies. Horizontal dashed lines indicate the overall mean pLDDT for each oligomer. The comparison shows that the prediction quality for monomeric subunits is highly consistent between the two assemblies.

The predicted PaCysE structure was superimposed with StCysE (PDB ID 8i09) to highlight similarities between the two orthologs (Figure S3), obtaining a Root Mean Square Deviation (RMSD) of 0.70 Å. As predicted from the sequence alignment, the catalytic core represents the only conserved region between the two orthologs, whereas both the N- and C-terminal regions differ substantially. PaCysE presents a C-terminal extension, absent in StCysE, which contains two cysteine residues (Cys223 and Cys234) that are predicted to be in close proximity, potentially enabling the formation of a stabilizing disulfide bond (Figure S3). Since CysE activity represents a central node in cysteine metabolism, their presence may be related to a hypothetical redox sensing mechanism. In fact, cysteine biosynthesis regulators in other bacterial species are known to regulate their activity by Cys residue oxidation. In *Staphylococcus aureus*, the master regulator of cysteine metabolism CymR presents a Cys residue sensitive to intracellular redox state [63]; in analogy, the transcriptional factors OhrR in *Bacillus subtilis* [64], and OxyR in *E. coli* [65] are regulated by Cys oxidation state, raising the question if a similar sensing mechanism might control PaCysE activity. In addition, PaCysE lacks four N-terminal helices, which in StCysE participate in the trimer–trimer interaction surface stabilizing the hexameric assembly. The absence of this structural element in PaCysE could therefore suggest a different oligomeric organization in this isoform.

The predicted assembly was analyzed using PRODIGY (PROtein binDIng enerGY prediction, https://wenmr.science.uu.nl/prodigy/ (accessed on 1 April 2026) [66,67]) to obtain a quantitative estimate of quaternary interactions stability. While the monomer–monomer interactions within the trimer are extremely stable, the predicted affinity of the trimers within the hexamer is markedly lower and falls within the high micromolar range (Table 1). In contrast, PRODIGY analysis of the hexameric interface in StCysE indicates a dissociation constant in the picomolar range (Table 1), supporting the formation of a stable hexamer for this isoform.

Analysis of the electrostatic surface potential (Figure 5D) indicates that the putative intertrimer interface is largely uncharged. In this context, comparison with *S. aureus* CysE (SaCysE) is particularly informative. At the sequence level, both SaCysE and PaCysE lack the same N-terminal region and are therefore classified as truncated forms. Consistently, SaCysE has been predicted to adopt a trimeric assembly [7]. Notably, it also displays a comparable electrostatic surface profile, characterized by a largely neutral interfacial region (Figure 5D). Taken together, these observations would support the idea that, in PaCysE, the formation of the hexamer assembly may be less favorable, and therefore potentially less populated under physiological conditions.

To determine whether the trimer indeed represents the predominant species, we used mass photometry; the analysis revealed the absence of a discrete oligomeric state, evidencing rather a coexistence of trimers (76 ± 23 kDa) and hexamers (186 ± 21 kDa), with the presence of higher molecular weight species, likely corresponding to aggregates (Figure S4). This finding, unexpected based on the *in silico* prediction, suggests that PaCysE may in principle adopt different, transient oligomeric states in vivo, warranting further structural investigations.

#### 2.2.2. Enzymatic Activity

The catalytic mechanism proposed for CysE involves a catalytic triad located at the protomer–protomer interfaces within the trimer, composed of His158, Asp143 (*E. coli* numeration) and the substrate L-Ser. The His residue deprotonates the L-Ser hydroxyl group, promoting the formation of an oxyanion intermediate with the acetyl moiety of AcCoA—stabilized by the imidazole ring of His193—the collapse of which generates OAS and CoA [12,68].

**Figure 6:**
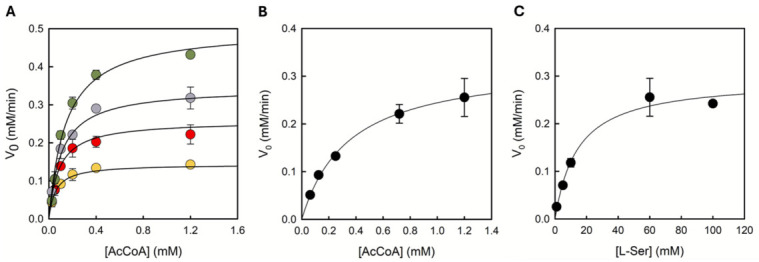
**Catalytic activity of PaCysE.** (**A**) The dependence of the initial rate on AcCoA concentration at varying L-Ser concentrations (2.5 mM, yellow; 5.8 mM, red; 10 mM, grey; 30 mM, green) was determined using the direct assay monitoring absorbance at 232 nm in buffer A (20 mM Na_2_HPO_4_, 85 mM NaCl, 1 mM EDTA, pH 7.0), with an enzyme concentration of 52 nM. The data were fitted to Equation (1) (see Materials and Methods), giving K_m,AcCoA_ = 0.18 ± 0.02 mM, K_m,L-Ser_ = 8.7 ± 1.1 mM, and k_cat_ = 208 ± 13 s^−1^. The dependence of the initial rate on AcCoA (**B**), at a fixed L-Ser concentration (60 mM), and on L-serine (**C**), at a fixed AcCoA concentration (1.2 mM), was determined using the indirect assay in the presence of DTNB as a reporter, in buffer A. The enzyme concentration was 52 nM. The data were fitted to the Michaelis–Menten equation, yielding K_m_^app^_,AcCoA_ = 0.35 ± 0.04 mM, K_m_^app^_,L-Ser_ = 14.0 ± 8.0 mM, and k_cat_^app^ values of 105 ± 7 s^−1^ and 95 ± 3 s^−1^, respectively.

The catalytic parameters of PaCysE were determined using a direct assay already applied to other CysE orthologs [13,69]); we collected a series of dependences of the initial rate of the reaction on AcCoA concentration keeping the concentration of L-Ser constant between 2.5 and 30 mM. Data were fitted globally to the equation for a random-order mechanism [13,70] (Figure 6A). Kinetic parameters are reported in Table 2. We obtained K_m,AcCoA_ = 0.18 ± 0.02 mM, K_m,L-Ser_ = 8.7 ± 1.1 mM and k_cat_ = 208 ± 13 s^−1^. These kinetic parameters suggest some interesting differences with respect to the EcCysE ortholog; while the K_m_ for AcCoA is comparable, the K_m_ for L-Ser is one order of magnitude higher, with the absence of substrate inhibition observed in other bacterial CysEs [13]. In addition, the value of K_D,L-Ser_, 1.01 ± 0.93 mM (Table 2) indicates that the already-observed strong negative substrate-binding synergism [13] is even more pronounced on PaCysE, with L-Ser that binds preferentially to the free enzyme rather than to the AcCoA–enzyme complex. To what extent these differences in catalytic activity are linked to specificities of the full-length and truncated forms of CysE is presently unknown, since the reports on truncated isoforms are presently limited to the orthologs of *M. tuberculosis* [35,71] and *S. aureus* [72] which are not entirely consistent and make use of the indirect assay based on DTNB that may affect the accuracy of the determined parameters (*vide infra*).

#### 2.2.3. Regulation

**Figure 7:**
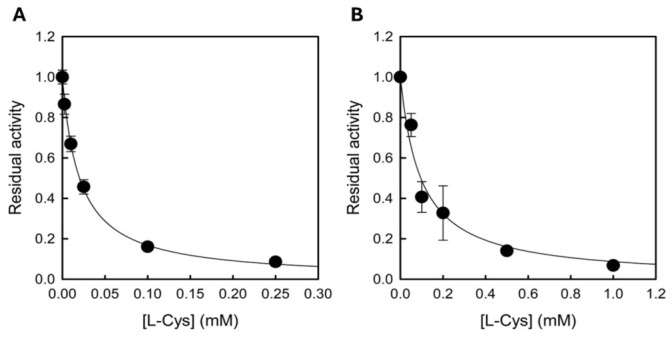
**Dependence of the residual activity of PaCysE on the concentration of L-Cys, in the presence of 250 μM AcCoA and either 6 mM (A) or 40 mM (B).** The reaction is carried out in buffer A with 53 nM PaCysE at 20 °C using the direct assay. Lines through data points are the fitting to Equation (2) with IC_50_ of 20 ± 1 μM and 96 ± 14 μM for the left and right panel, respectively.

Among the several checkpoints that regulate its biosynthesis, the feedback control exerted by L-Cys on CysE is one of the most relevant. Structural studies which obtained CysE crystal structures in the presence of its ligands demonstrated that L-Cys can inhibit the enzymatic activity by occupying the L-Ser binding site, as witnessed by the shared contacts in the holo crystal structures [10,12,18,33]. Together with a direct competition with L-Ser, L-Cys binding promotes the position rearrangement of CysE C-terminal tails, which partially occupy the AcCoA binding site (LβH loop), thereby reducing its apparent affinity for the enzyme [10]. Since the C-terminal region of the *P. aeruginosa* enzyme is more extended than those of other isoforms, it cannot be excluded that a different rearrangement occurs, involving different portions of the tail and leading to a different regulation of the feedback inhibition. We thus performed two titrations of PaCysE in the presence of two L-Ser concentrations, i.e., 6 and 40 mM, that are below and above the K_m_ value, respectively. The IC_50_ values calculated from the fitting are 20 ± 1 μM and 96 ± 14 μM in the presence of 6 and 40 mM L-Ser, respectively (Figure 7). This result indicates that the activity of PaCysE is inhibited by L-Cys in a competitive way; indeed, the apparent affinity of the amino acid decreases with increasing L-Ser concentration. On the other hand, the IC_50_ values are at least 100-fold higher with respect to the one measured on the *E. coli* ortholog under comparable conditions [13]. This finding indicates that PaCysE might be less susceptible to feedback inhibition by L-Cys; nevertheless, due to the competitive nature of this inhibition, the increase in the K_m_ for L-Ser with respect to the *E. coli* ortholog might partially compensate for L-Cys decreased affinity. In the case of the *E. histolytica* enzyme, which also exhibits a high K_i_ value for L-Cys, the authors suggest that the absence of allosteric binding of the C-terminal region near the active site, which buries the AcCoA binding site, may be a major determinant of the lower affinity observed with respect to the *E. coli* and *S.* Typhimurium enzymes. In this view, inhibition would not simply reflect competitive binding, but would also involve a conformational change, consistent with the structural rearrangements required for slow-binding inhibition [18]. Interestingly, the Phe256 residue (*H. influenzae* numeration) is reported to be accommodated in the adenine moiety binding pocket; this residue, conserved also in *S.* Typhimurium and *E. coli* sequences (Phe260), is not present in the PaCysE sequence (Figure 1).

**Figure 8:**
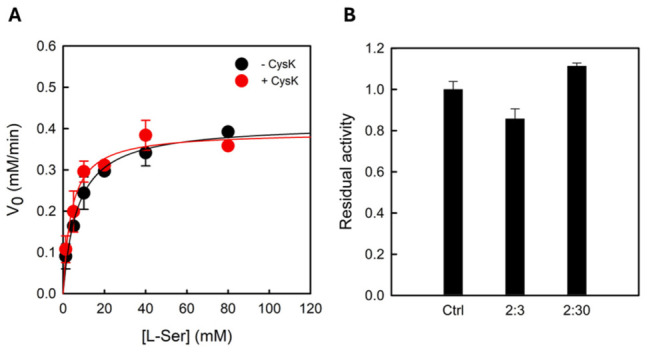
**Monitoring PaCysE-PaCysK complex formation.** (**A**) Dependence of the initial reaction rate on the concentration of L-Ser, in the presence of 250 μM AcCoA, in the absence (black dots) and presence (red dots) of 265 nM PaCysK. The reaction is carried out in buffer A with 53 nM PaCysE at 20 °C using the direct assay. Lines through data points provide visual guidance. (**B**) Residual activity of PaCysK in the presence of a 2:3 and 2:30 molar ratio of PaCysE.

Another notable modulator of CysE activity in *E. coli* is CysK, that, by forming a complex with the enzyme alleviates inhibition by L-Ser, results in an apparently higher catalytic efficiency. On the other hand, complex formation leads to almost complete inhibition of CysK [73]. We investigated this aspect by measuring the Michaelis–Menten dependences of PaCysE in the presence and absence of PaCysK (Figure 8A). The effect, if any, is negligible. The same observation holds true when measuring the activity of PaCysK, either in the presence of a stoichiometric amount of PaCysE as in the CSC or in a molar excess (Figure 8B). These results agree with the observation that the PaCysE sequence lacks a C-terminal isoleucine residue, the anchor point for complex formation in CSC-forming organisms discovered so far (Figure 1, [74,75,76]).

### 2.3. CysE Inhibitors Testing

#### 2.3.1. *In Vitro* Activity Assay

**Figure 9:**
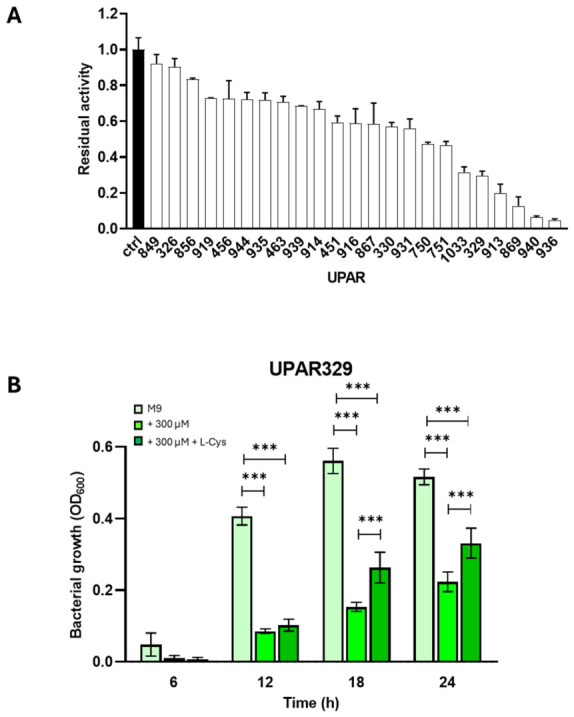
**Activity of CysE inhibitory compounds.** (**A**). *In vitro* screening of potential inhibitors on PaCysE. Compounds were tested at 0.1 mM in the presence of 10 mM L-Ser, 0.15 mM AcCoA, 53 nM CysE, 5% DMSO, 1 mM DTNB in buffer A at 20 °C. Control experiments were carried out in the absence of compounds, while maintaining the presence of 5% DMSO. (**B**). Activity of UPAR329 on *P. aeruginosa*. Growth of *P. aeruginosa* WT was monitored in M9 supplemented or not with 300 µM UPAR329 in the presence or absence of 40 µg/mL L-Cys. Data points represent the mean of three independent biological replicates, each performed at least in duplicates, and error bars indicate the corresponding standard deviations (SD). Statistical analysis was performed using one-way ANOVA with Tukey’s multiple comparisons test. Differences between M9 and M9 supplemented with UPAR329, with or without L-Cys, were performed at all the indicated time points using one-way ANOVA with Tukey’s multiple comparisons test and were considered statistically significant if *p* < 0.001 (***).

Our group has previously developed inhibitors targeting CysE orthologs StCysE and EcCysE showing low micromolar dissociation constants [32,36]. These compounds originated from a virtual screening campaign performed on StCysE, which led to the identification of a set of chemically diverse inhibitors subsequently validated experimentally for enzyme inhibition. Considering the overall sequence conservation of CysE among bacterial species, we reasoned that part of this chemical space could retain activity against PaCysE. Based on this rationale, a subset of 23 compounds was selected and tested at a fixed concentration of 0.1 mM (Figure 9A and Table S2). The selection was guided by both their inhibitory activity against StCysE and the need to ensure chemical diversity. The selected compounds share common heterocyclic and aromatic cores, with variations in the peripheral substituents that allow for a preliminary structure–activity comparison. This approach allowed us to evaluate whether inhibitors previously identified for StCysE retained activity against PaCysE and to explore the extent to which inhibitory profiles are conserved across orthologous enzymes. The direct assay used to calculate catalytic parameters (Figure 6A and Table 2) is not suitable for the screening of inhibitors because the totality of the molecules contains an aromatic moiety that would absorb in the UV region of the spectrum, potentially interfering with the signal of thioester bond at 232 nm. We thus determined the catalytic parameters using a previously validated indirect assay [9,77,78] by fitting the dependence of the rate on the concentration of one substrate while keeping the other substrate constant (Figure 6B,C). We obtained K_m_^app^_,AcCoA_ = 0.20 ± 0.09 mM and K_m_^app^_,L-Ser_ = 14 ± 8 mM; the k_cat_^app^ values were identical within the experimental error (about 100 s^−1^), an indication that the fixed substrate reached almost saturation for both conditions. The parameters obtained with this assay indicate an overall decrease in the measured catalytic activity with respect to the direct one (decrease in k_cat_ values and increase in K_m_ values) that confirms an already-observed trend [9] that can be ascribed to interference of DTNB in the assay due to a non-covalent interaction of the molecule with the enzyme [70]. Despite this, the assay has been successfully adapted for the screening of the compound library against StCysE [36]. We used this assay at subsaturating substrate concentrations to screen the compound library (Figure 9A).

Six compounds showed residual activity below 50% at 100 μM, i.e., their IC_50_ values are below 100 μM. For these compounds, the IC_50_ values were determined (Figure S5) and are reported in Table 3. Compound UPAR913 was not investigated further, as it is structurally similar to UPAR940, with both molecules bearing a nitro moiety, as for the most potent compounds identified in the initial screening. As reported in Table 3, these molecules display activity in the low micromolar range, with compound UPAR936 showing an IC_50_ of 1.43 ± 0.5 µM, and compounds UPAR940 and UPAR869 exhibiting comparable potency. While this feature may contribute to their activity, nitro functionalities are widely recognized as structural alerts due to their association with safety and metabolic liabilities. [79]. Moreover, it is considered a suboptimal feature from a medicinal chemistry perspective and will require replacement with more suitable functionalities in future optimization efforts. However, given their low micromolar activity, these compounds were retained for further evaluation at this preliminary stage and included in the subsequent *P. aeruginosa* growth assays. Within the subset, compound UPAR329 emerged as the most promising candidate due to its chemical properties. A comparable profile is observed for compound 1033, which, despite showing a more moderate activity (IC_50_ = 67.2 ± 16.5 µM) and limited solubility in the assay conditions, still represents a relevant scaffold within the series.

Based on these preliminary results, the five compounds underwent further investigation in *P. aeruginosa* cultures. They represent suitable starting points for hit-to-lead optimization, where structural modifications can be explored to improve potency while maintaining favorable chemical properties.

#### 2.3.2. Effect of the CysE Inhibitory Compounds on *P. aeruginosa*

The best hit compounds identified against PaCysE (i.e., UPAR936, 940, 869, 329 and 1033) were tested on *P. aeruginosa* WT (Figure 9B). Assay concentrations were set at 300 µM for compounds UPAR329 and UPAR869, which is, in both cases, much higher than the *in vitro* IC_50_ values. For compounds UPAR936, UPAR940, and UPAR1033, the assay concentration was reduced to 100 µM due to solubility limitations, nevertheless reaching a tenfold excess with respect to IC_50_, except for UPAR1033 (less than twofold). To this end, *P. aeruginosa* WT were cultured in M9 medium, or M9 in the presence of CysE inhibitors with or without 40 µg/mL L-Cys. Results demonstrated that, among the tested compounds, UPAR329 exhibited the highest activity, significantly inhibiting bacterial growth by more than 70% at 12 h and 18 h post-inoculation, and by ca. 60% at 24 h (Figure 9B). L-Cys supplementation only partially restored growth in the presence of UPAR329, suggestive of a likely minor off-target effect. UPAR936 and UPAR940 showed intermediate effects, reducing bacterial growth by ca. 50% at 12 h post-inoculation; however, this inhibition diminished over time (Figure S6A). Similarly, UPAR1033 caused an approximately 40% reduction in growth at 12 h, with less effect at later time points (Figure S6A). The weaker activity of these compounds with respect to UPAR329 might also be attributed to the lower concentration used in the assay. UPAR869 exhibited a slight activity at all times tested, suggesting that the compound may not efficiently enter the cell.

Unfortunately, L-Cys supplementation failed to rescue growth in the presence of all compounds, except UPAR329, where a small yet significant increase in growth was observed at 18 and 24 h post-inoculation. These results suggest that the observed effects of the other compounds likely reflect a general antibacterial activity rather than selective inhibition of L-Cys biosynthesis. To further investigate this possibility, the activity of the CysE inhibitory compounds was assessed in rich medium (i.e., LB), where *de novo* cysteine synthesis is not essential. Interestingly, under these conditions, none of the UPAR compounds tested affected the growth of *P. aeruginosa* WT, at all the time points (see Figure S6B), thus arguing against a general anti-*Pseudomonas* activity, at least in this experimental setting.

## 3. Materials and Methods

### 3.1. Cloning

The sequence of the *P. aeruginosa* gene PA3816 (UniProt entry Q9HXI6) was synthesized and optimized for the expression in *E*. *coli* host and cloned into a modified pASK-IBA3plus vectors (IBA Lifesciences, Göttingen, Germany) to obtain a construct presenting an N-terminal Strep-tag.

### 3.2. Protein Expression and Purification

PaCysE was expressed in *E. coli* BL21 Tuner cells grown in LB medium at 37 °C, in the presence of 100 µg/mL ampicillin. When the culture reached OD_600_ = 0.6, protein expression was induced by adding 0.2 µg/mL anhydrotetracycline at 37 °C under agitation. After 3 h incubation, cells were harvested by centrifugation, washed once in phosphate-buffered saline (PBS) buffer, and stored at –20 °C until further use. For protein purification, cells were resuspended in lysis buffer (100 mM Tris, 150 mM NaCl, 1 mM EDTA, 10% glycerol, 0.01% Brij-35, pH 8.0), in the presence of 0.2 mM phenylmethylsulfonyl fluoride, 0.2 mM benzamidine, 1.5 µM pepstatin A, and 1 mg/mL lysozyme; after 45 min incubation at 4 °C under stirring, cells were sonicated in ice by 5 s pulses interspersed with 1 min pauses. The lysate was then incubated for 30 min at 4 °C in the presence of 3.33% streptomycin sulphate (*w*/*V*) under stirring and afterwards centrifuged for 30 min at 18,000× *g* to obtain the soluble fraction. PaCysE was purified by affinity chromatography on a benchtop column on Strep-Tactin^®^ XT 4Flow^®^ resin (IBA Lifesciences GmbH, Göttingen, Germany); the resin was washed with 3-bed column volumes of lysis buffer in the presence of 10 mM MgCl_2_, and the protein was eluted by 1× BXT buffer, pH 8.0 (IBA Lifesciences GmbH, Göttingen, Germany), in the presence of 1 mM tri(2-carboxyethyl)phosphine. The fractions containing PaCysE were pooled and diafiltered in the lysis buffer by 30 MWCA RC Amicon^®^ Ultra Centrifugal Filter (Merck Millipore, Darmstatd, Germany) to remove biotin and concentrate the protein. PaCysE final concentration was calculated from the absorption spectrum (Figure S2) at 280 nm (ε = 45,950 M^−1^ cm^−1^, MW 30,280 Da, determined by ProtParam (Expasy, https://web.expasy.org/protparam/ (accessed on 6 November 2024)); the protein solution was flash frozen in liquid nitrogen and stored at –80 °C until further use.

### 3.3. Activity Assays

The enzymatic activity of PaCysE was assessed using continuous spectrophotometric assays. Catalytic parameters and IC_50_ for L-Cys were determined by a direct assay that monitors the decrease in absorbance at 232 nm, corresponding to the consumption of AcCoA [13]. Inhibitors screening and inhibitors IC_50_ values were measured using an indirect DTNB-based assay, in which 2,2′-dinitro-5,5′-dithiobenzoic acid reacts with the free thiol group of coenzyme A released during the PaCysE-catalyzed reaction, producing a mixed disulfide (CoA–TNB) and the chromophoric TNB^−^ anion, which can be monitored at 412 nm. Reactions were carried out at 20 °C in buffer A (20 mM Na_2_HPO_4_, 85 mM NaCl, 1 mM EDTA, pH 7.0) using quartz cuvettes with different pathlengths according to the absorbance range of measurement. Reactions were initiated by the addition of PaCysE, and absorbance changes were recorded using a Cary 4000 UV–vis spectrophotometer (Agilent Technologies, Santa Clara, CA, USA). Initial velocities (v_0_) were determined from the linear portion of the progress curves and converted to µM·min^−1^ using the appropriate molar extinction coefficients: ε_412_ nm = 14,000 M^−1^ cm^−1^ for TNB and ε_232_ nm = 4440 M^−1^ cm^−1^ for AcCoA.

The dependence of v_0_ on AcCoA or L-Ser concentration at saturating concentration of the other substrate was determined using the indirect DTNB-based assay and fitted to the Michaelis–Menten equation to determine the apparent K_M_ and k_cat_.

The dependence of v_0_ on both L-Ser and AcCoA concentrations was measured using the direct assay. L-Ser was varied from 2.5 to 30 mM while AcCoA was varied from 25 μM to 1.2 mM, and the resulting initial velocities were globally fitted to Equation (1) for a random-order kinetic mechanism [13,80]:

(1) $$v_{0}=\frac{V_{max}\cdot \left[AcCoA\right]\cdot [L-Ser]}{{K_{D,Ser}\cdot K_{m,AcCoA}+K}_{m,Ser}\cdot \left[AcCoA\right]+K_{m,AcCoA}\cdot \left[L-Ser\right]+\left[AcCoA\right]\cdot \left[L-Ser\right]}$$

where *V*_max_ is the reaction rate at saturating substrates concentration; *K*_m,Ser_ is the Michaelis constant for L-Ser; K_m,AcCoA_ is the Michaelis constant for AcCoA; and *K*_D,Ser_ is the dissociation constant of L-Ser from the unliganded enzyme.

The inhibitory effect of L-Cys on CysE activity was evaluated by measuring the initial velocity at a fixed AcCoA concentration (0.25 mM), L-Ser concentrations of 6 mM and 40 mM and in the presence of varying L-Cys concentrations. Data were fitted to Equation (2) to calculate IC_50_ values:

(2) $$\frac{v_{i}}{v_{0}}=\frac{{IC}_{50}}{1+[I]}$$

where v_i_ is the initial velocity in the presence of a given L-Cys concentration, v_0_ is the initial velocity in the absence of L-Cys, IC_50_ is the L-Cys concentration that gives a relative activity of 0.5 and [I] is the L-Cys concentration.

The effect of CysE on the activity of CysK was determined using an ion-selective electrode (Unisense H2S microsensor Type I, SULF-NP-403877, Aarhus, Denmark), following the protocol described by Martedì et al. [41] with small adjustments. Enzymatic assays were conducted in a final volume of 1.5 mL in 100 mM Hepes, pH 7.0, containing a final concentration of either 3.5 or 35 nM CysK, 52.5 nM CysE, 35 nM BSA, 1 mM OAS and 100 μM bisulfide at 25 °C. The reaction was initiated by the addition of a mixture of the two enzymes. Bisulfide was generated directly in the reaction mixture using Na_2_S solutions. The concentration of bisulfide was calculated from the concentration of Na_2_S and then plotted versus time. Initial velocities were obtained by subtracting to the reaction time course the slope of the initial phase.

### 3.4. Compound Synthesis

All compounds tested in this study were previously reported and their synthesis has been described in detail in the original publications [32,78]. Chemical structures and purity of the compounds were confirmed as reported in the corresponding references, including full characterization by NMR and mass spectrometry.

The unpublished compound 3-chloro-6-methyl-n-(5-methylthiazol-2-yl)benzo[b]thiophene-2-carboxamide (UPAR916) was synthesized as follows, based on Scheme S1. Under nitrogen atmosphere, 1,1′-Carbonyldiimidazole (1.5 eq.) was added to a solution of 3-chloro-5-methylbenzo[b]thiophene-2-carboxylic acid (1 eq.) in dry DMF, and the mixture was stirred for 1 h at room temperature. 5-methylthiazol-2-amine (1 eq.) was then added and the reaction mixture stirred at room temperature overnight. Reaction was quenched with H_2_O, extracted with ethyl acetate (3 × 10 mL) and the organic layers were dried over anhydrous sodium sulphate, filtrated and concentrated under reduced pressure to yield a crude product that was purified by flash column chromatography eluting dichloromethane/methanol (99:1). The title compound was isolated as a white powder in 71% yield.

^1^H NMR (400 MHz, DMSO) δ 2.3 (s, 3H), 2.5 (s, 3H), 7.2 (s, 1H), 7.4 (d, j = 8, 1H), 7.8 (d, j = 8, 1H), 7.9 (s, 1H), 12.8 (s, 1H). ^13^C NMR 162.1, 161.8, 141.6, 137.8, 135.4, 133.2, 132.9, 129.9, 124.9, 123.5, 122.7, 120.5, 21.7, 11.8.

### 3.5. Compounds Screening

Compounds were dissolved in 100% dimethylsulfoxyde (DMSO) to prepare stocks at the concentrations indicated in Table S2. They were tested at 100 μM for their ability to inhibit CysE activity by the indirect DTNB-based assay, at 20 °C in buffer A in the presence of 5% DMSO. Control reference assays were performed in the presence of 5% DMSO, in the absence of compounds. The most promising hits underwent IC_50_ calculation by varying inhibitor concentration at 20 °C in buffer A in the presence of 10 mM L-Ser, 150 μM AcCoA, and 5% DMSO. Data were fitted to Equation (2).

### 3.6. Circular Dichroism

After buffer-exchange into 20 mM potassium phosphate (pH 7.0) using Amicon ultracentrifugal diafiltration devices (RC, 30 kDa, Merck-Millipore, Darmstandt, Germany), circular dichroism spectra were collected with a Jasco™ J-1500 spectropolarimeter (JASCO Corporation, Tokyo, Japan) equipped with a Peltier thermostatic unit set at 20 °C. Spectra were collected over the wavelength range of 260–190 nm in continuous scanning mode with a bandwidth of 2 nm, a data pitch of 0.5 nm, an integration time of 8 s, and a scanning speed of 50 nm min^−1^. Three consecutive scans were recorded for each sample and averaged. Secondary structure estimation was performed by using the Bestsel server (https://bestsel.elte.hu/index.php (accessed on 15 February 2026)) [81].

For the estimation of the melting temperature, far-UV CD signal changes at 222 nm were monitored as a function of temperature from 30 °C to 100 °C, with a ramp rate of 5 °C per minute. Data were fitted to Equation (3).

(3) $${\left[\theta \right]}_{MRW}={\left[\theta \right]}_{MRW}^{0}+\frac{f}{1+e^{\frac{T-T_{m}}{k}}}$$

where [θ]_MRW_ is the mean residue ellipticity at 222 nm, ${\left[\theta \right]}_{MRW}^{0}$ is an offset, f is the amplitude of the thermal transition, T is the temperature in °C, T_m_ is the melting temperature and k is the slope of the phase.

[θ]_MRW_ was calculated as

(4) $${\left[\theta \right]}_{MRW}=\frac{\theta \cdot 100\cdot MRW}{c\cdot l}$$

where θ is the ellipticity in mdeg, MRW is the mean residue weight, *c* is the concentration in mg/mL and *l* is pathlength in cm.

### 3.7. Structural Prediction and Analysis

The structure of PaCysE was predicted using AlphaFold 3 [62] on the high-performance computing (HPC) cluster of the University of Parma. Structural visualization and analysis, including surface representation and electrostatic potential mapping, were performed using ChimeraX [82]. Binding affinities (ΔG and KD) of protein complexes were estimated using PRODIGY [66,67], available at https://wenmr.science.uu.nl/prodigy/ (accessed on 1 April 2026).

### 3.8. Mass Photometry

Mass Photometry measurements were performed on 1.5H glass cover slips, cleaned with sequential washes in 2-propanol and MilliQ water, using a Refeyn TwoMP mass photometer (Refeyn Ltd., Oxford, UK). The 100 nM samples of the PaCysE were diluted 1:10 in 20 mM potassium phosphate buffer, pH 7.0, and a 60 s movie was recorded using Refeyn AquireMP software (release 2024 R2). Movies were subsequently analyzed using the Refeyn DiscoverMP software (release 2024 R2) to quantify the protein binding events. Protein molecular weights were determined by comparison against a movie recorded on the Refeyn MassFerence P1 mass calibrant solution, prepared as per the manufacturer’s instructions.

### 3.9. Bacterial Strains and Culture Conditions

Strains and plasmids used in this study are listed in Table S3. Bacteria were routinely grown in LB broth with good aeration (shaking at 180 rpm) or in LB-Agar (LA). When required, antibiotics were added to the media at the following concentrations: 100 μg/mL ampicillin (Ap), 20 μg/mL nalidixic acid (Nal), 10 μg/mL chloramphenicol (Cm), 12.5 μg/mL tetracycline (Tc), 25 μg/mL kanamycin (Km) for *E. coli* and 100 μg/mL Tc for *P. aeruginosa*. When required, media were supplemented with 40 µg/mL L-Cys.

### 3.10. Chemically Defined Media and Inhibitor Assessment

All media and solutions were prepared using deionized, double-distilled water (ddH_2_O). In this study, two media were used: (i) a chemically defined minimal medium M9 [83]; and (ii) a modified version of M9 in which the S-source was substituted with thiosulfate (Na_2_S_2_O_3_) at final concentration of 0.5 mM and MgSO_4_ was replaced with an equimolar concentration of MgCl_2_ [41]. The ability of *P. aeruginosa* WT and its isogenic Δ*cysE* mutant to grow in the presence of different S-sources was investigated by monitoring bacterial growth at 600 nm (OD_600_) over time. Strains were pre-cultured in LB at 37 °C with 180 rpm shaking, then bacterial cells were washed once in saline, diluted to OD_600_ = 0.001 in the appropriate growth medium and dispensed in a 96-well microplate (200 µL/well), and OD_600_ was measured over time using a microplate reader (SPARK 10M TECAN^®^, Tecan Group Ltd., Männedorf, Switzerland).

The five UPAR compounds were dissolved in DMSO and stored at −20 °C until use. Stock solutions of UPAR329 and UPAR869 were prepared at 100 mM, whereas UPAR936 and UPAR940 were prepared at 10 mM, and UPAR1033 at 50 mM. When required, M9 medium was supplemented with the inhibitory compounds at final concentrations of 300 μM for UPAR329 and UPAR869, and 100 μM for UPAR936, UPAR940, and UPAR1033. Equimolar concentrations of DMSO alone were included in the experiments as negative controls.

### 3.11. Construction of Plasmids for Molecular Cloning

Standard genetic manipulations were performed according to Sambrook et al. [83]. FastDigest restriction enzymes were purchased from Thermo Fisher Scientific (Waltham, MA, USA) and used in accordance with the instructions provided by the manufacturer. For the deletion of *cysE* in the *P. aeruginosa* PAO1 chromosome a 913 bp fragment overlapping the ATG of *cysE* and a 981 bp fragment overlapping the TAA of *cysE* were amplified by PCR using the genomic *P. aeruginosa* PAO1 DNA as template and the primer couples *cysE*UPFW: 5′-TGCTCTAGAAACCGATGGCGTTTGAAG-3′/*cysE*UPRV: 5′-CCGGAATTCGAACATCAGTCGTCACTC-3′ and *cysE*DWFW: 5′-CGCGAATTCAATCCGGCCGCGTAATGC-3′/*cysE*DWRV: 5′-GGCGGTACCTTGTGCTCGATCTTCGAG-3′, respectively. The PCR-amplified upstream and downstream regions of *cysE* gene were digested with XbaI-EcoRI and EcoRI-KpnI, respectively, and then subcloned into the corresponding sites of pBluescript II SK^−^ (pBS), yielding plasmids pBS∆*cysE* before being cloned XbaI-KpnI into the final suicide vector pME3087 [84], yielding plasmids pME∆*cysE*. The latter were then introduced into *P. aeruginosa* PAO1 by triparental mating, using the helper strain *E. coli* HB101/pRK2013, as previously described [85,86]. The resulting *P. aeruginosa* strains ∆*cysE* carried an in-frame deletion in the gene of interest. The deletion was also confirmed by PCR and subsequent sequencing.

To complement the *cysE* mutation, a DNA fragment containing the *cysE* gene with its own promoter region was PCR amplified from the *P. aeruginosa* PAO1 genome using primer couple *cysE*_compl_FW: 5′-CGGGGTACCCGATGGAGCGGAAAATCAGT-3′/*cysE*_compl_RV: 5′-CCGGAATTCAGGGCGCTACATGCTAG-3′. The PCR product was then digested with KpnI and EcoRI and cloned into the corresponding sites of the shuttle vector pME6031 [87], giving pME*cysE* plasmid. This plasmid was then introduced into *P. aeruginosa* ∆*cysE* to genetically complement the previously generated mutation.

### 3.12. Phenotypic Characterization of the Deletion Mutant

#### 3.12.1. Measurement of Intracellular ROS Levels

CysE-mediated reactive oxygen species (ROSs) generation in *P. aeruginosa* was determined by using the redox-sensitive, cell-permeant fluorescent dye 2′,7′-dichlorodihydrofluorescein diacetate (H_2_DCFDA) (Sigma-Aldrich, Saint Louis, MO, USA) as previously described [88], with some modifications. *P. aeruginosa* strains were pre-cultured in LB for ca. 16 h, then refreshed in LB supplemented or not with 40 µg/mL L-Cys to reach mid-log exponential phase (OD_600_ ≈ 0.6). Cells were then collected by centrifugation, washed with PBS, and resuspended in PBS at an OD_600_ of about 1. Bacteria were incubated with 10 μM H_2_DCFDA (stock solution 10 mM, dissolved in DMSO) in the dark at 37 °C for 30 min. Aliquots (200 µL) of each bacterial suspension were dispensed in a 96-well black-microtiter plate. Fluorescence emissions from DCFDA were measured at 480 nm excitation and 535 nm emission wavelengths using a microplate reader (SPARK 10M TECAN^®^, Tecan Group Ltd., Männedorf, Switzerland) and normalized to the OD_600_ of each sample. PBS with 10 μM H_2_DCFDA was used as a blank.

#### 3.12.2. Growth and Biofilm Analysis

Bacterial cultures were grown for 16 h in LB, then washed and diluted to an OD_600_ of 0.05 in 200 µL in M63 medium supplemented or not with 40 µg/mL L-Cys in 96-well microtiter plates and grown for 24 h at 37 °C. Growth was measured spectrophotometrically (OD_600_) at the end point, and biofilm formation was quantified following the protocol described by O’Toole [54]. Briefly, planktonic cells were removed, and the attached cells were gently washed with ddH_2_O, air dried, and stained for 15 min with 250 µL of 0.1% crystal violet (CV) in ddH_2_O. Wells were then gently washed twice with ddH_2_O, and the surface-associated dye was dissolved in 250 µL of 30% acetic acid for 15 min at RT. The OD_550_ of the CV eluate was measured in a microplate reader (SPARK 10M TECAN^®^, Tecan Group Ltd., Männedorf, Switzerland).

#### 3.12.3. Pyocyanin Quantification

Pyocyanin production was quantified following the protocol described in [89]. Briefly, 5 mL of culture grown in LB in glass flasks medium was extracted with 3 mL of chloroform. The chloroform phase containing pyocyanin was subsequently re-extracted with 1 mL of 0.2 N HCl, producing a pink to deep red aqueous phase. The absorbance of the acidified solution was measured spectrophotometrically at 520 nm. Pyocyanin concentration, expressed as micrograms (µg) of pyocyanin per mL of supernatant, was calculated by multiplying the OD_520_ value by the conversion factor 17.072.

#### 3.12.4. Swarming Assay

Swarming motility assays were performed on solid Nutrient Broth (NB) No2 medium (Difco), containing 0.6% (*w*/*V*) agar and 20% (*w*/*V*) D-glucose. Stationary-phase cultures grown in LB were spotted (10 µL) onto agar plates and allowed to dry prior to incubation. Swarming was observed after 18 h of incubation at 37 °C. The images are representative of three independent experiments with similar results.

#### 3.12.5. *Galleria mellonella* Infection

Experiments were performed using 30 larvae per trial. For control conditions, both no injection (survival control) and larvae injected with 10 µL sterile saline (negative control) were included. Experiments were conducted up to 48 h. For *P. aeruginosa* infection, stationary-phase bacterial cultures grown in LB were washed, normalized to an OD_600_ = 1, and subsequently diluted 1:10 to achieve the desired bacterial concentration. Larvae were injected with 10 µL of a 10^3^ CFU/mL suspension and incubated at 37 °C for 48 h. After injection, the larvae were incubated for 48 h at 37 °C. Survival and macroscopic appearance were monitored every 6 h post-injection. Larvae that did not respond to gentle prodding using forceps were scored as dead and removed from the experiment. Survival was expressed as a percentage. The experiments were performed on two independent occasions. Survival curves were analyzed in GraphPad Prism 8.0 (GraphPad Software, Boston, MA, USA) using the log-rank (Mantel–Cox) test, and *p* values < 0.05 were considered statistically significant.

## 4. Conclusions

In this work, we have identified and validated PA3816 as the major serine acetyltransferase of *P. aeruginosa* PAO1 and demonstrated that its product, PaCysE, plays a central role not only in cysteine biosynthesis but also in the control of several physiological traits linked to bacterial fitness and virulence. The combination of genetic, biochemical and structural analyses highlights PaCysE as a distinctive member of the CysE family and, to our knowledge, provides the first integrated biochemical and physiological characterization of a member of the truncated CysE isoforms [7]. Deletion of *cysE* resulted in a marked cysteine auxotrophy under the conditions tested, both when sulfate and thiosulfate were provided as sulfur sources, indicating that PaCysE catalyzes a committed and non-redundant step of *de novo* cysteine biosynthesis in *P. aeruginosa*. This is an important point also in comparison with previous observations on *cysK* and *cysM* mutants, which were not auxotrophic, consistent with the partial functional overlap of the two OASS isoforms [41]. In contrast, the auxotrophy of the Δ*cysE* strain indicates that no alternative SAT activity is expressed at levels sufficient to compensate for the loss of PaCysE in the tested conditions, despite the presence of another putative acetyltransferase in the genome. This places CysE upstream of the branch point catalyzed by CysK/CysM and identifies it as a more stringent bottleneck for pathway function. It is also worth considering that, in many bacterial species, the product of OAS activates, after isomerization to NAS, the transcription of the cysteine regulon, with a mechanism still poorly investigated in *P. aeruginosa* that involves the regulator CysB [90]. The phenotypic characterization of the Δ*cysE* mutant further showed that impairment of cysteine biosynthesis has pleiotropic consequences extending well beyond amino acid auxotrophy. The recovery of Δ*cysE*-associated phenotypes by exogenous cysteine supplementation or genetic complementation strongly supports the conclusion that PaCysE contributes to redox homeostasis and virulence-associated behaviors mainly through its control over cysteine availability.

From a biochemical standpoint, PaCysE displays kinetic and regulatory properties that distinguish it from the canonical full-length enterobacterial orthologs. In particular, the high K_m_ for L-Ser and the reduced susceptibility to feedback inhibition by L-Cys indicate that PaCysE operates under a different regulatory regime. At first sight, weaker inhibition by L-Cys might suggest a looser feedback control; however, this effect should be interpreted together with the lower apparent affinity for L-Ser. Since intracellular concentrations of L-Ser and L-Cys are expected to lie in a similar low-micromolar range [91,92] and [L-Ser] is known to fluctuate substantially depending on central carbon metabolism, the elevated K_m_ may render enzyme activity particularly sensitive to changes in substrate availability. PaCysE also apparently lacks functional interaction with CysK. Unlike the well-characterized CSC of *E. coli* and other bacteria, PaCysE did not measurably affect PaCysK activity, nor was its own activity significantly altered by PaCysK. This behavior is consistent with the absence of the canonical C-terminal isoleucine required for CysK binding [93]. CysK binding may have evolved in some bacteria as an additional regulatory layer; in contrast, PaCysE seems to have dispensed with this regulatory strategy, possibly because its distinctive structural and functional features support a different balance between catalytic activity, stability, and feedback control. These features include its lower sensitivity to cysteine inhibition, its quaternary assembly, and the presence of a C-terminal extension, all of which point to a substantially different interaction and regulatory network [94]. In EcCysE, the interaction with the partner CysK elicits long-distance structural communication and rearrangements [21]; it is possible that in PaCysE the elongated C-terminus plays a specific function in the overall conformational rearrangement caused by the interaction with still undiscovered interactors or effectors. These findings also highlight important implications for antimicrobial targeting. The pronounced auxotrophic phenotype of the Δ*cysE* mutant, together with its defects in oxidative stress response and virulence-associated traits, identifies CysE as a highly vulnerable node in *P. aeruginosa* physiology, particularly under nutrient-limited conditions. Since CysE functions upstream of both CysK and CysM, its inhibition would be expected to block *de novo* cysteine biosynthesis more effectively than targeting either downstream sulfhydrylase alone. This makes CysE an especially attractive candidate for impairing bacterial adaptation when cysteine availability is restricted. This idea is supported by previous studies showing that CysE inhibition can increase antibiotic susceptibility in *E. coli* [33] and *M. tuberculosis* [27] and by our findings of a reduced virulence of *P. aeruginosa* Δ*cysE* in the *G. mellonella* animal model.

By defining the physiological role, catalytic features, and druggability of PaCysE, our results broaden the current view of bacterial CysE diversity and provide a foundation for future studies aimed at understanding how truncated SAT isoforms are regulated and how this metabolic vulnerability may be exploited for antibacterial strategies. More broadly, this work highlights how inhibition of the same enzymatic function can produce markedly different physiological and pathological outcomes even in closely related bacterial species. A deeper understanding of both the shared and species-specific contributions of cysteine biosynthetic enzymes to virulence-related processes is therefore timely and may help advance the pharmacological exploitation of this pathway.

## Figures and Tables

**Table 1:** **Binding affinities (ΔG, K_D_) and interfacial contacts predicted by PRODIGY** [66] **for PaCysE oligomeric states and the StCysE hexamer (PDB ID 8i09).** * Results represent the average values calculated for each monomer relative to the other two.

Protein–Protein Complex	ΔG (kcal/mol)	K_D_ (M)
PaCysE monomer–monomer *	−27.9	3.3 × 10^−21^
PaCysE trimer–trimer	−4.9	2.5 × 10^−4^
StCysE trimer–trimer	−17.7	1 × 10^−13^

**Table 2:** **Kinetic parameters of PaCysE.** The parameters obtained by the indirect assay were calculated in the presence of a fixed concentration of 60 mM L-Ser or 1.2 mM AcCoA and varying the second substrate.

Assay	K_m,AcCoA_ (mM)	K_m,L-Ser_ (mM)	K_D,L-Ser_ (mM)	k_cat_ (s^−1^)	k_cat_/K_m,AcCoA_ (M^−1^·s^−1^)	k_cat_/K_m,L-Ser_ (M^−1^·s^−1^)
Direct	0.18 ± 0.02	8.7 ± 1.1	1.01 ± 0.93	208 ± 13	(1.2 ± 0.2) · 10^6^	(2.4 ± 0.3) · 10^4^
Indirect	0.35 ± 0.04	14.0 ± 8.0	/	105 ± 7	(3.0 ± 0.4) · 10^5^	(7.5 ± 4.3) · 10^3^

**Table 3:** **Structures and IC_50_ values of the best hits selected by the *in vitro* activity assay.**

UPAR	Structure	IC_50_ (μM)	Reference
936	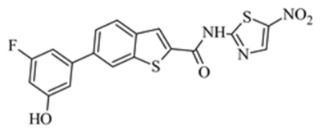	1.43 ± 0.5	[32]
869	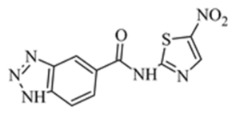	6.9 ± 0.9	[32]
940	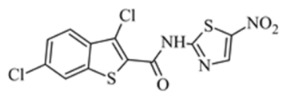	8.0 ± 1.3	[32]
329	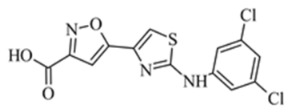	29.8 ± 5.6	[78]
1033	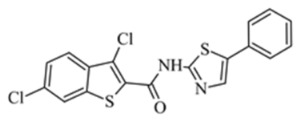	67 ± 17	[32]

## Data Availability

The raw data supporting the conclusions of this article will be made available by the authors on request.

## Supplementary Materials

The following supporting information can be downloaded at https://www.mdpi.com/article/10.3390/ijms27115091/s1.

## Abbreviations

The following abbreviations are used in this manuscript:

AcCoA	Acetyl-coenzyme A
CD	Circular dichroism
CFU	Colony forming units
CoA	Coenzyme A
CSC	Cysteine synthase complex
CysE	Serine acetyltransferase
CysK	*O*-acetylserine sulfhydrylase A
CysM	*O*-acetylserine sulfhydrylase B
DCF-DA	2′,7′-dichlorodihydrofluorescein diacetate
ddH_2_O	Double-distilled water
DMSO	dimethylsulfoxyde
DTNB	5,5′-dithiobis-(2-nitrobenzoic acid)
FU	Fluorescence units
GSH	Glutathione
H_2_DCFDA	2′,7′-dichlorodihydrofluorescein diacetate
L-Cys	L-cysteine
L-Ser	L-serine
LB	Luria–Bertani medium
M63	Minimal medium M63
M9	Minimal medium M9
NB	Nutrient broth medium
NAS	*N*-acetylserine
OAS	*O*-acetylserine
OD_600_	Optical density at 600 nm
OASS	O-acetylserine sulfhydrylase
PBS	Phosphate-buffered saline
pLDDT	Predicted Local Distance Difference Test
RMSD	Root mean square deviation
ROS	Reactive oxygen species
RT	Room temperature
SAT	Serine acetyltransferase
TNB	2-nitro-5-thiobenzoate
WT	Wild type
Δ*cysE*	*cysE* deletion mutant
