# Supplementary material for: Serine Acetyltransferase from Pseudomonas aeruginosa: Distinctive Features, Pleiotropic Roles, and Therapeutic Potential
Source: Int J Mol Sci. 2026 Jun 4;27(11):5091. doi: 10.3390/ijms27115091 (PMC13256707; doi:10.3390/ijms27115091)
Supplement: Supplementary file 1 [file ijms-27-05091-s001.zip › ijms-4317449-supplementary.pdf]

Supplementary Materials

Serine acetyltransferase from *Pseudomonas aeruginosa*: distinctive features, pleiotropic roles, and therapeutic potential

Francesco Guggino[1#], Sarah Hijazi[2#], Rebecca Martedì[2], Valeria Buoli Comani[3], Jole Maria Lucia D'Angelo[4], Omar De Bei[5], Giannamaria Annunziato[4], Marco Pieroni[4], Gabriele Costantino[4], Stefano Bettati[1,3,5], Marialaura Marchetti[5*], Emanuela Frangipani[2*], Barbara Campanini[3,4]

1 Interdepartmental center Siteia, University of Parma, Parma, Italy; francesco.guggino@studenti.unipr.it (F.G.); stefano.bettati@unipr.it (S.B.)

2 Department of Biomolecular Sciences, University of Urbino Carlo Bo, Urbino, Italy; emanuela.frangipani@uniurb.it (E.F.)

3 Interdepartmental center Biopharmanet-TEC, University of Parma, Parma, Italy; valeria.buolicomani@unipr.it (V.B.C.); barbara.campanini@unipr.it (B.C.)

4 Department of Food and Drug, University of Parma, Parma, Italy; jolemarialucia.dangelo@unipr.it (J.M.L.D.A.)

5 Department of Medicine and Surgery, University of Parma, Parma, Italy; omar.debei@unipr.it

* Correspondence: marialaura.marchetti@unipr.it (M.M.); emanuela.frangipani@uniurb.it (E.F.)

\# Equal contribution

Table S1. Comparison of secondary structure content obtained by deconvolution of CD spectra and by analysis of the AlphaFold 3 structure generated in this work. Deconvolution was performed with BestSel [1], while secondary structure elements were calculated on the predicted structure using KCD [2].

Model	Alpha helix (%)	Strand (%)	Other (%)
CD (pre-melting)	27.1	22.6	50.3
CD (post-melting)	20.8	26.4	52.8
AlphaFold 3 prediction	29.6	25.9	44.4

 https://doi.org/10.3390/10.3390/ijms27115091

Table S2. Structures of inhibitors, concentration of stock solutions in 100% DMSO, literature reference (in brackets; numbers in parentheses are the numbers attributed to the compounds in the original publication), and enzymatic residual activity in the screening in the presence of 0.1 mM compound.

UPAR	Structure	Stock (mM)	Reference	Residual Activity (%)
936	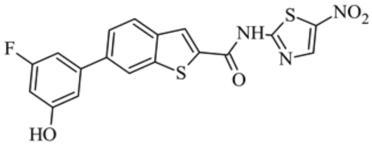	10	[3] (16)	4.7
940	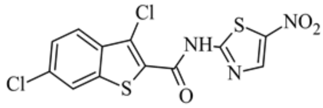	10	[3] (14)	6.3
869	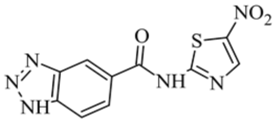	100	[3] (11)	12.6
913	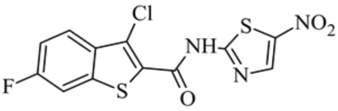	50	[3] (13)	19.9
329	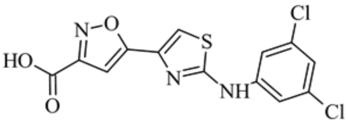	100	[4](20)	29.6
1033	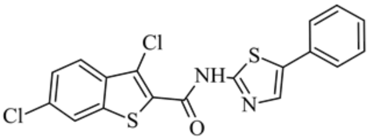	50	[3] (68)	31.5
751	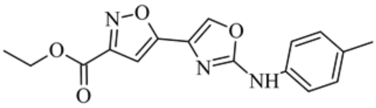	20	[4] (19)	46.6
750	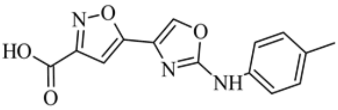	50	[4] (18)	47.3
931	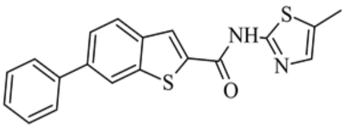	10	[3] (48)	56
330	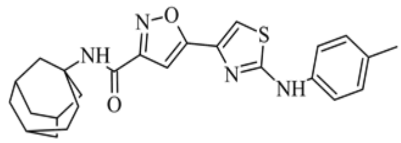	100	[4] (10)	56.9
867	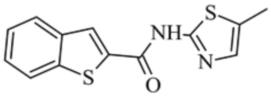	50	[3] (41)	58.4

916	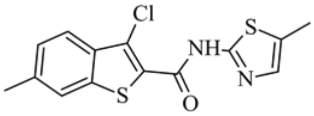	10	This study	58.7
451	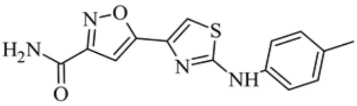	100	[4] (11)	59.3
914	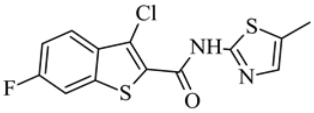	10	[3] (43)	66.8
939	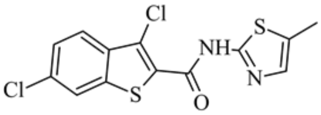	10	[3] ()	68.4
463	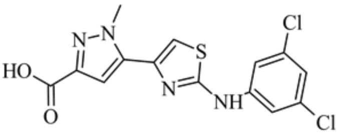	100	[4] (17)	70.6
935	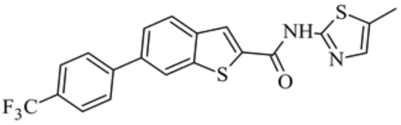	10	[3] (46)	71.8
944	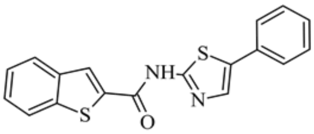	25	[3] (67)	72
456	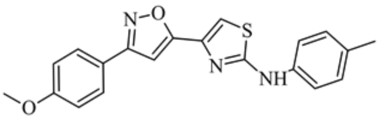	50	[4] (9)	72.4
919	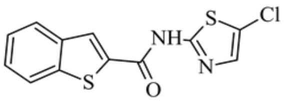	50	[3] (63)	72.7
856	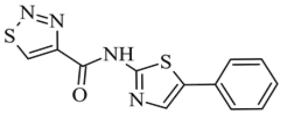	20	[3] (64)	83.3
326	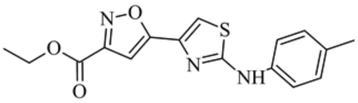	100	[4] (14)	90.3
849	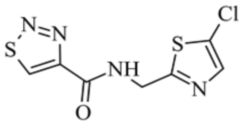	100	[3] (61)	92.1

Table S3. Bacterial strains and plasmids used in this study.

Strain or plasmid	Genotype and/or relevant characteristics	References or source
Strains		
P. aeruginosa strains		
PAO1	ATCC 15692 (wild type, prototroph)	American Type Culture Collection (Manassas, VA, USA)
cysE mutant	PAO1 Δ*cysE*	This study
*cysE*pMEΔ*cysE*	PAO1 Δ*cysE* harboring pME6031 derivative carrying the coding sequence of *cysE* with its own promoter	This study
E. coli strains		
DH5α	*recA1 endA1 hsdR17 supE44 thi-1 gyrA96 relA1* Δ(*lacZYA-argF*)U169 (φ80d*lacZ*ΔM15) F^{-}, Nal^{R}	[5]
HB101	*proA2 hsdS20* (r_b m_b) *recA13 ara-14 lacY1 galK2 rpsL20 supE44 xyl-5 mtl-1* F^{-}	[5]
Plasmids		
pRK2013	Helper plasmid; Tra^{+} Km^{R}	[6]
pME3087	Suicide vector for allelic replacement; ColE1 replicon, Tc^{R}	[7]
pMEcysE	pME3087 carrying a 1,975 bp deletion in the *cysE* gene	This study
pBluescript II SK^{-} (pBS)	Cloning vector, ColE1 replicon, Ap^{R}	Stratagene (La Jolla, CA, USA)
pBSΔ*cysE*	pBS carrying a 1,975 bp deletion in the *cysE* gene, Ap^{R}	This study
pME6031	Broad host range plasmid; Tc^{R}	[8]
pME*cysE*	pME6031 derivative carrying the coding sequence of *cysE* with its own promoter	This study

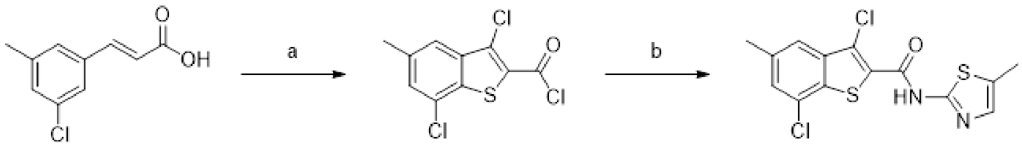

Scheme S1. Reagents and conditions for 3-chloro-6-methyl-n-(5-methylthiazol-2-yl)benzo[b]thiophene-2-carboxamide, (UPAR916) synthesis. a) $SOCl_2$, Pyr, DMF, 130 °C, 5 h, 76%; b) 5-methylthiazol-2-amine, dioxane, reflux, 3 h, 68%.

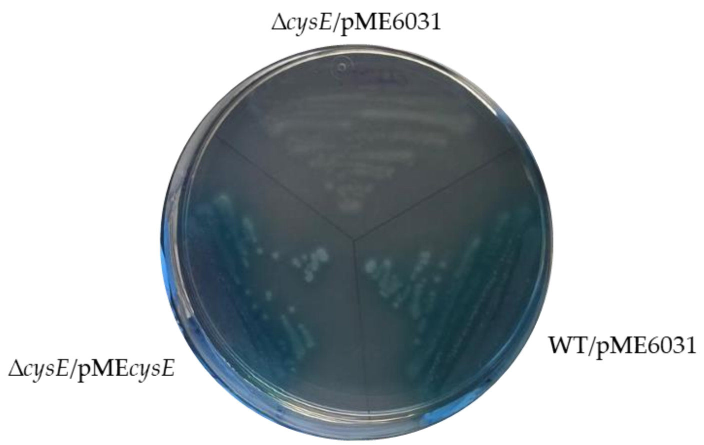

Figure S1. Growth of *P. aeruginosa* WT, *ΔcysE* and *ΔcysE*/pME*cysE* on Nutrient-Broth agar (NA). The three strains were streaked on a NA plate into separate sectors and incubated at 37 °C for 16 h.

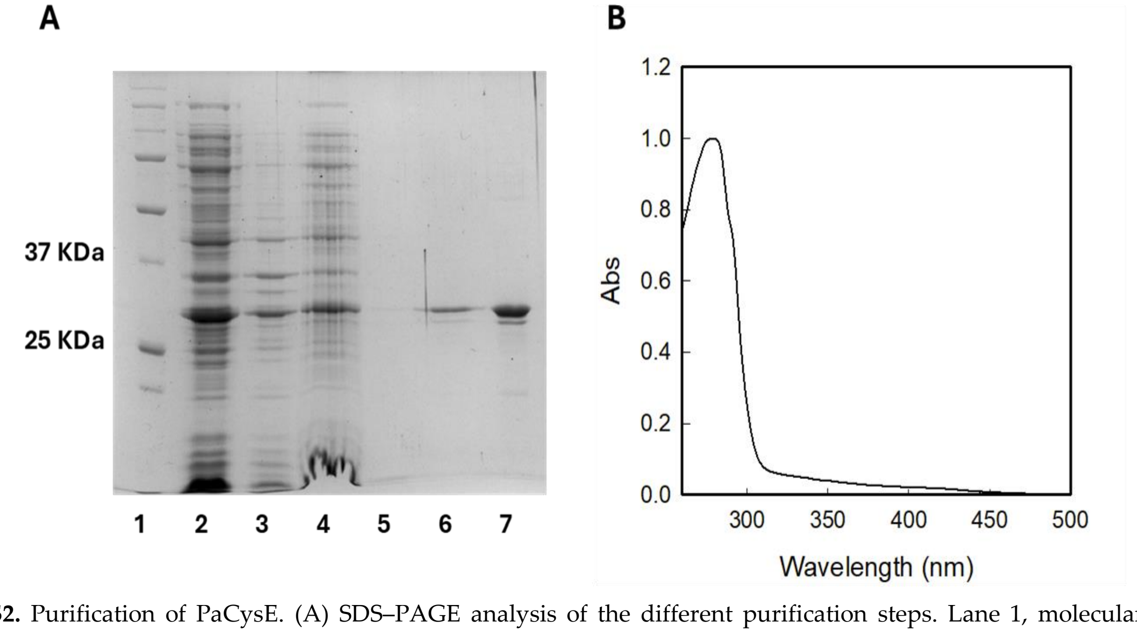

Figure S2. Purification of PaCysE. (A) SDS–PAGE analysis of the different purification steps. Lane 1, molecular weight marker; lane 2, soluble fraction; lane 3, insoluble fraction; lane 4, flow-through; lane 5, wash fraction; lane 6, eluted protein; lane 7, protein after concentration. (B) UV-visible absorption spectrum of the purified protein after concentration.

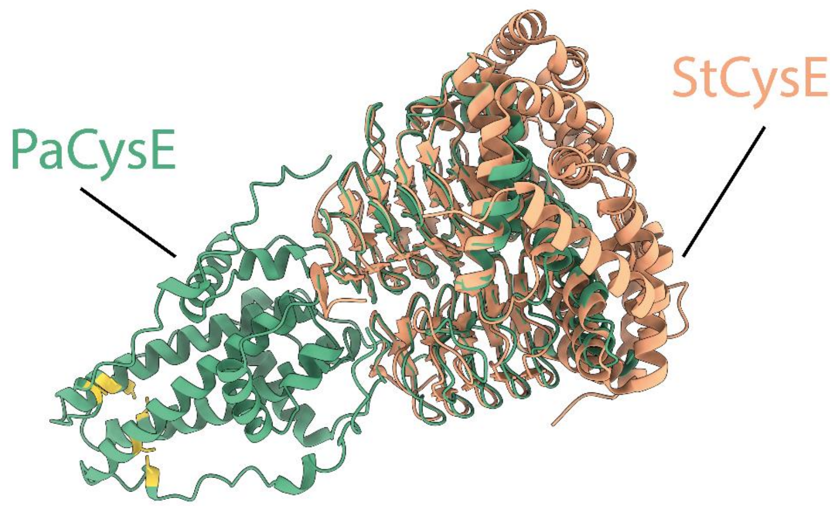

Figure S3. Superimposition of the predicted structure of PaCysE (green) with the crystallographic structure of StCysE (PDB 8i09, chains L, J and K) (orange), yielding an RMSD of 0.70 Å over 156 aligned atom pairs. The cysteine residues on PaCysE C-terminal domain are shown in yellow.

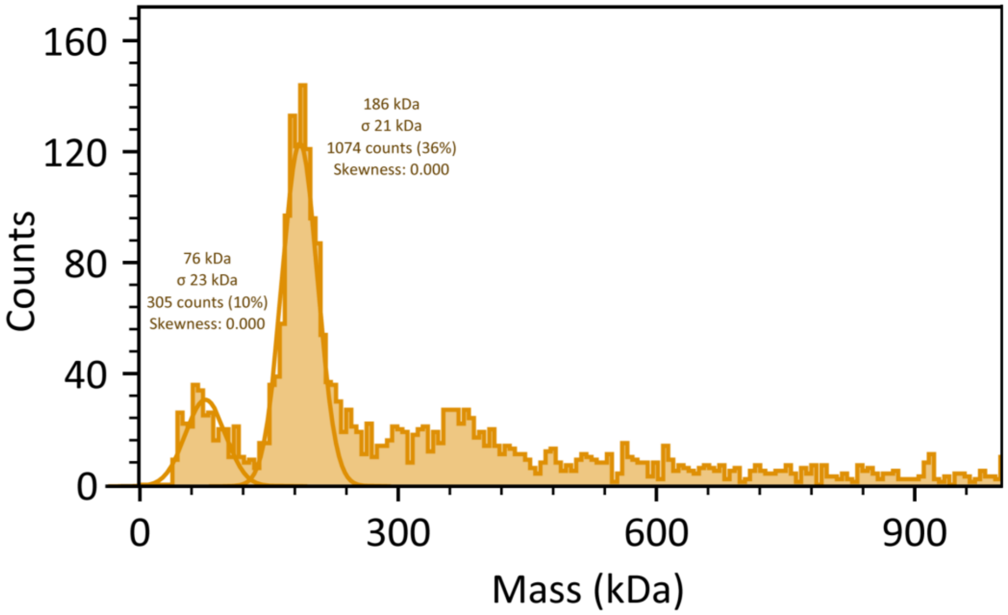

Figure S4. Mass photometry analysis of PaCysE. Distinct peaks correspond to different oligomeric species, with mean molecular weights indicated. Measurements were performed in duplicate. Gaussian fitting of the population distributions is shown. Populations exhibiting skewness > 0.5 were excluded from further analysis.

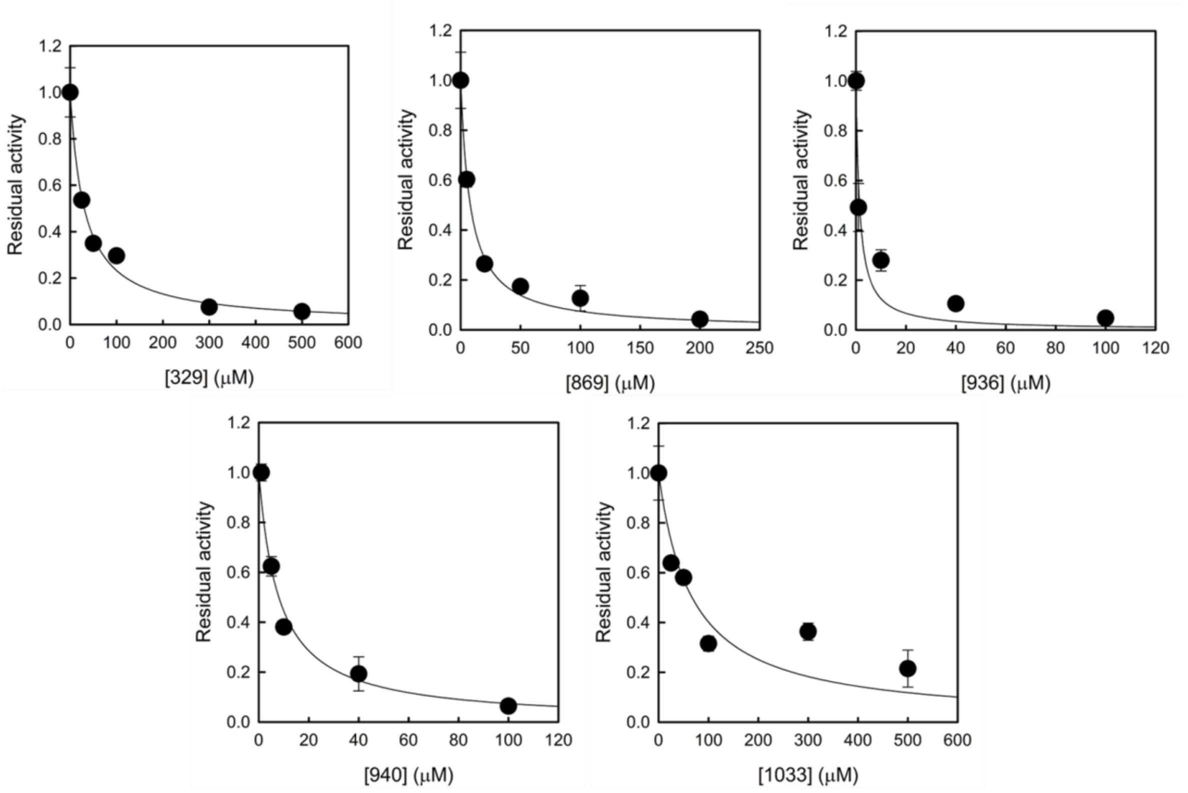

Figure S5. IC_{50} determination for the five selected inhibitors against CysE. Compounds were tested at increasing concentrations in the presence of 10 mM L-Ser, 0.15 mM AcCoA, 53 nM CysE, 5% DMSO, 1 mM DTNB in buffer A at 20 °C. The dependence of the relative activity on compounds concentration was fitted to Equation 2 with IC_{50} values of 30 ± 6 μM, 1.3 ± 0.5 μM, 67 ± 17 μM and 8 ± 1, 7 ± 1 μM for compounds UPAR 329, 936, 1033, 940 and 869 respectively. Data points represent the mean ± std dev of at least two independent experiments.

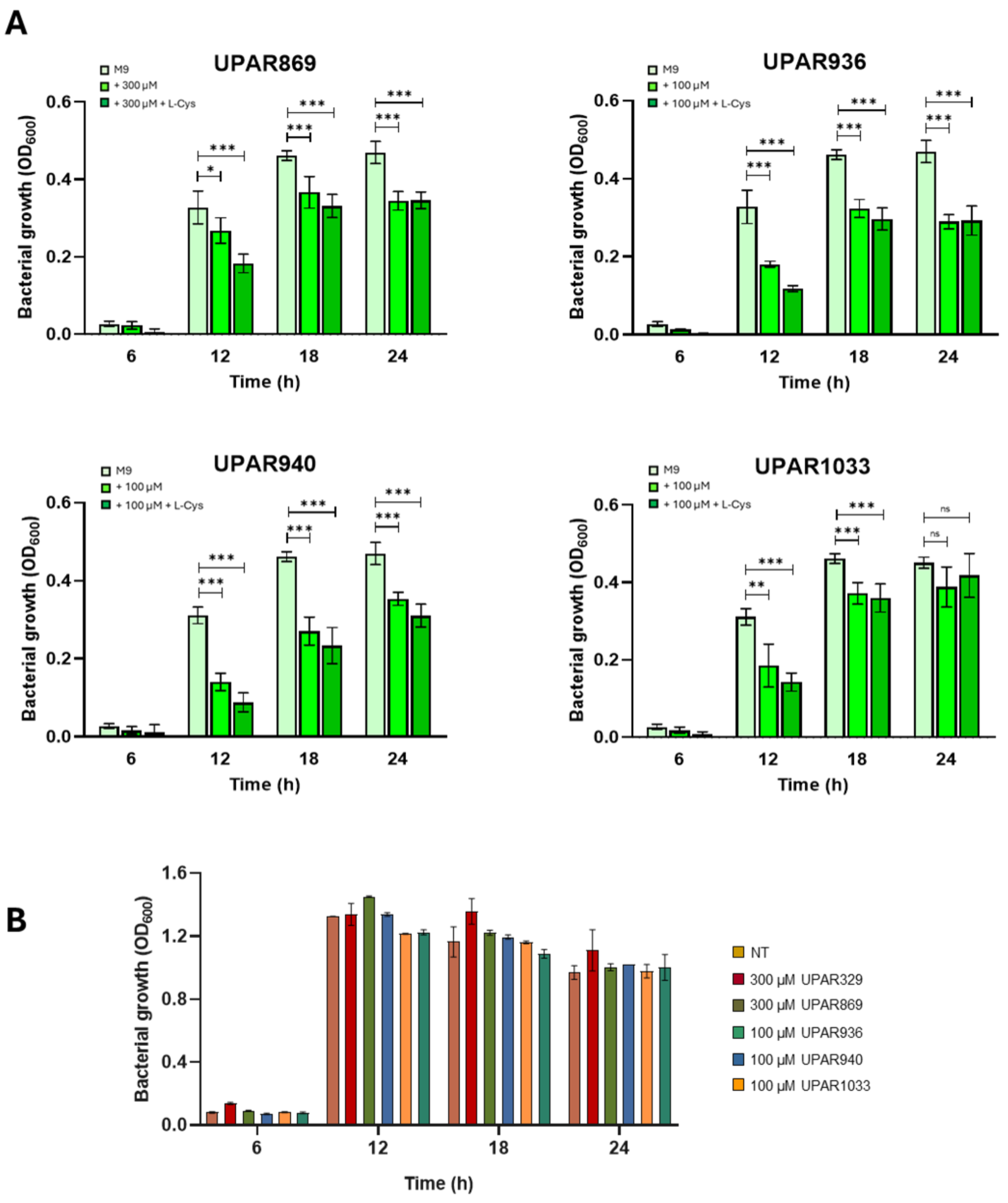

Figure S6. Activity of CysE inhibitory compounds on *P. aeruginosa* in M9 (**A**) or in the nutrient-rich medium LB (**B**). Growth of *P. aeruginosa* WT was monitored in both media supplemented or not with UPAR compounds at the indicated concentrations. Data points represent the mean of at least two independent biological replicates, each performed at least in duplicate. Statistical analysis was performed using one-way ANOVA with Tukey's multiple comparisons test. Differences between M9 and M9 supplemented with UPAR, with or without L-Cys, were performed at all the indicated time points and were considered statistically significant if ***$P < 0.001$, **$P < 0.01$; *$P < 0.05$, or not significant (ns) $P \geq 0.05$.

References

1. Micsonai, A.; Wien, F.; Kernya, L.; Lee, Y.-H.; Goto, Y.; Réfrégiers, M.; Kardos, J. Accurate Secondary Structure Prediction and Fold Recognition for Circular Dichroism Spectroscopy. *Proceedings of the National Academy of Sciences* **2015**, *112*, E3095–E3103, doi:10.1073/pnas.1500851112.
2. Jacinto-Méndez, D.; Granados-Ramírez, C.G.; Carbajal-Tinoco, M.D. KCD : A Prediction Web Server of Knowledge-based Circular Dichroism. *Protein Science* **2024**, *33*, e4967, doi:10.1002/pro.4967.
3. Pavone, M.; Raboni, S.; Marchetti, M.; Annunziato, G.; Bettati, S.; Papotti, B.; Marchi, C.; Carosati, E.; Pieroni, M.; Campanini, B.; et al. Exploring the Chemical Space around N-(5-Nitrothiazol-2-Yl)-1,2,3-Thiadiazole-4-Carboxamide, a Hit Compound with Serine Acetyltransferase (SAT) Inhibitory Properties. *Results in Chemistry* **2022**, *4*, 100443, doi:10.1016/j.rechem.2022.100443.
4. Magalhães, J.; Franko, N.; Raboni, S.; Annunziato, G.; Tammela, P.; Bruno, A.; Bettati, S.; Armao, S.; Spadini, C.; Cabassi, C.S.; et al. Discovery of Substituted (2-Aminooxazol-4-Yl)Isoxazole-3-Carboxylic Acids as Inhibitors of Bacterial Serine Acetyltransferase in the Quest for Novel Potential Antibacterial Adjuvants. *Pharmaceuticals* **2021**, *14*, 174, doi:10.3390/ph14020174.
5. Sambrook, J.; Fritsch, E.F.; Maniatis, T. *Molecular Cloning: A Laboratory Manual*; 2nd. ed.; Cold Spring Harbor laboratory press: Cold Spring Harbor, 1989; ISBN 978-0-87969-309-1.
6. Ditta, G.; Schmidhauser, T.; Yakobson, E.; Lu, P.; Liang, X.-W.; Finlay, D.R.; Guiney, D.; Helinski, D.R. Plasmids Related to the Broad Host Range Vector, pRK290, Useful for Gene Cloning and for Monitoring Gene Expression. *Plasmid* **1985**, *13*, 149–153, doi:10.1016/0147-619X(85)90068-X.
7. Voisard, C.; Bull, C.T.; Keel, C.; Laville, J.; Maurhofer, M.; Schnider, U.; Défago, G.; Haas, D. Biocontrol of Root Diseases by *Pseudomonas Fluorescens* CHA0: Current Concepts and Experimental Approaches. In *Molecular Ecology of Rhizosphere Microorganisms*; O'Gara, F., Dowling, D.N., Boesten, B., Eds.; Wiley, 1994; pp. 67–89 ISBN 978-3-527-30052-5.
8. Heeb, S.; Itoh, Y.; Nishijyo, T.; Schnider, U.; Keel, C.; Wade, J.; Walsh, U.; O'Gara, F.; Haas, D. Small, Stable Shuttle Vectors Based on the Minimal pVS1 Replicon for Use in Gram-Negative, Plant-Associated Bacteria. *MPMI* **2000**, *13*, 232–237, doi:10.1094/MPMI.2000.13.2.232.
